# Real-Time Affinity Measurements of Proteins Synthesized in Cell-Free Lysate Using Fluorescence Correlation Spectroscopy

**DOI:** 10.1021/acs.analchem.4c05485

**Published:** 2025-04-30

**Authors:** Chao Liu, Steven A. Hoang-Phou, Congwang Ye, Emma J. Laurence, Matthew J. Laurence, Erika J. Fong, Nikki M. Hammond, B. Dillon Vannest, Nicholas N. Watkins, Ted A. Laurence, Matthew A. Coleman

**Affiliations:** 1 Biosciences and Biotechnology Division, Lawrence Livermore National Laboratory, Livermore, California 94550, United States; 2 Materials Engineering Division, Lawrence Livermore National Laboratory, Livermore, California 94550, United States; 3 Materials Science Division, Lawrence Livermore National Laboratory, Livermore, California 94550, United States

## Abstract

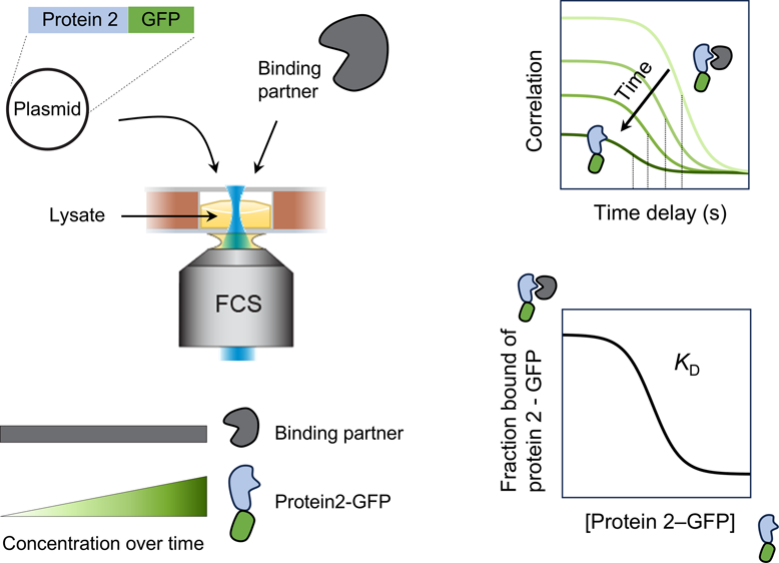

Rapid, high throughput measurements of biomolecular interactions are essential across medicine and bioscience. Traditional methods for affinity-screening proteins require a long and costly process involving cell-based expression, purification, and titration of multiple concentrations to arrive at a binding curve. In contrast, we have developed a fast and simple approach that yields a wealth of information about the expression of the protein and its binding characteristics, all in a “one-pot reaction” and done in under several hours without the need for protein purification. The method uses cell-free protein synthesis to produce the protein of interest in the presence of its binding partner while simultaneously using fluorescence correlation spectroscopy (FCS) to measure the increasing concentration of the protein and its binding to the binding partner. We characterize the sensitivity limits of this method by measuring the binding between the green fluorescent protein (GFP) and a low picomolar-affinity anti-GFP antibody and found that we can quantify *K*_D_ down to the high picomolar to low-nanomolar range. We further demonstrate the method in a potentially ultrahigh-throughput sample format in which FCS measurements are collected inside microcapsules. This work lays the foundation for a platform aimed at the production and in situ affinity screening of thousands of different proteins.

## Introduction

High throughput screening is critical for developing therapeutics for new and reemerging threats from pathogens.^1^ Strategies for increasing the efficiency and speed of this screening are valuable for testing candidate therapeutics, which may include libraries of compounds and computational designs.^2,3^ In this work, we developed a strategy for reducing the steps in the screening process. Conventionally, the production of proteins is separate from a screening step that measures interactions between proteins. Here we introduce a strategy in which we measure the binding affinity for protein–protein interactions simultaneously with the production of the proteins using fluorescence correlation spectroscopy (FCS). Importantly we combine FCS with coupled transcription and translation cell-free protein synthesis (“cell-free” for short) lysates, which enable the production of proteins encoded by plasmid DNA *in vitro*. We can assess the concentration of produced protein as it increases while also measuring binding to its binding partner in solution. With this strategy, proteins can be designed, synthesized, and screened efficiently, all in “one-pot reactions”, in which the entire process for each protein is completed in a single sample solution.

**Figure 1:**
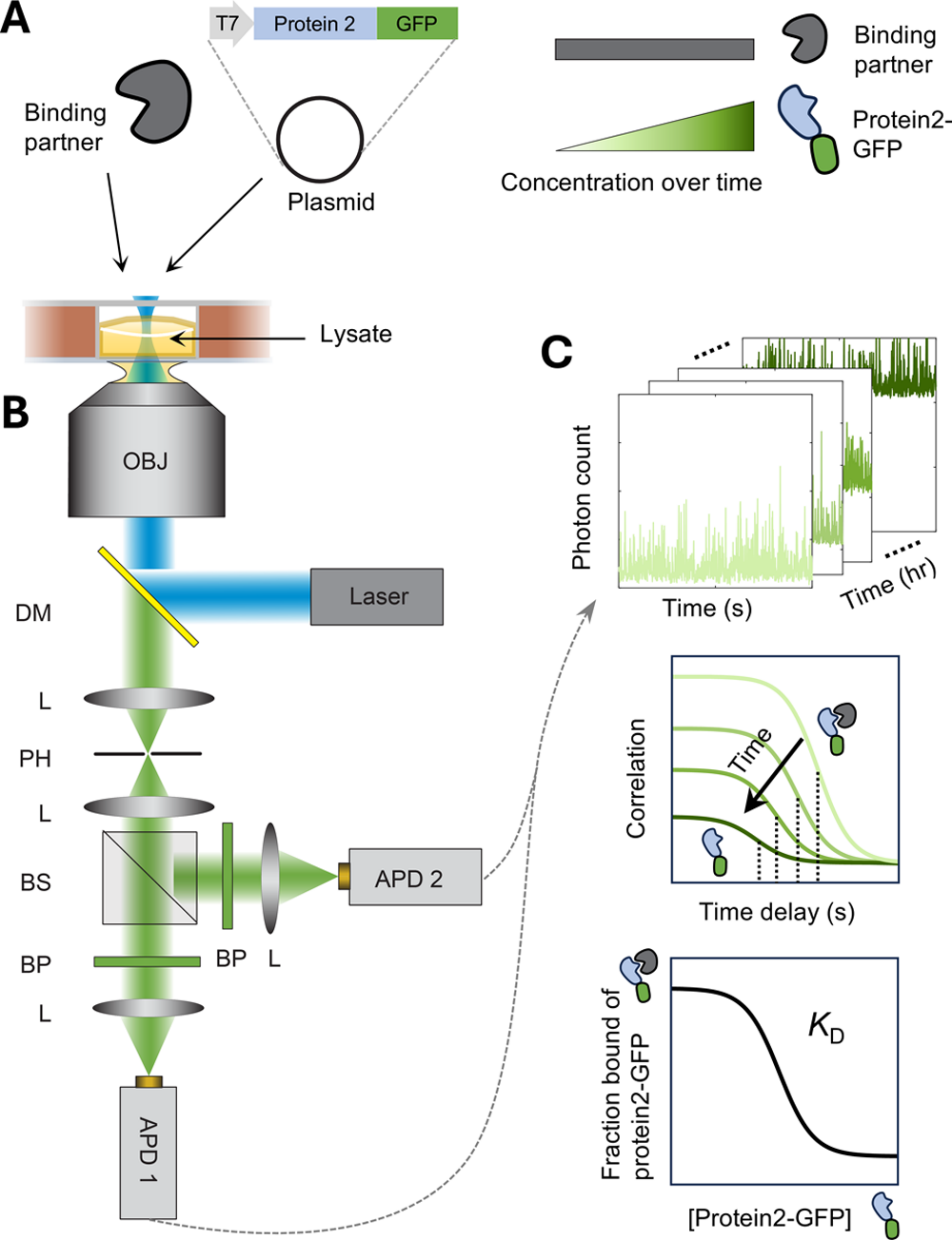
Experimental setup to measure binding of protein during cell-free synthesis by fluorescence correlation spectroscopy (FCS). (A) Reactions consist of the cell-free expression lysate, the protein of interest (“Binding partner”), and a plasmid encoding the fluorescently tagged second protein (“Protein2-GFP”) that is expected to bind to the Binding partner. Reaction solutions (∼6 μL) are incubated in sample wells between coverslips, keeping a layer of air above. The concentration of Protein2-GFP increases over time as the expression proceeds, while that of the Binding partner remains constant. (B) Samples are measured over time by FCS using a 488 nm excitation laser, a confocal pinhole (PH), and two avalanche photodiodes (APDs). OJB: objective. DM: dichroic mirror. L: lens. BS: beamsplitter. BP: bandpass filter. (C) Top: data consist of arrival times of each photon on the detectors, collected for different samples over several hours of protein synthesis. Middle: over time, calculated correlations are expected to shift down and left, as amplitude decreases due to increasing protein concentration while overall diffusion time decreases due to an excess of free Protein2-GFP over its Binding partner. Bottom: a two-species fit to correlations yields the fraction of Protein2-GFP bound and its concentration, producing a binding curve from which the binding affinity (*K*_D_) is determined.

FCS is sensitive to individual fluorescently labeled molecules diffusing in solution into and out of an open detection volume defined by a tightly focused laser beam and a confocal pinhole.^4−6^ Protein interactions can be measured by the increase in diffusion time due to the increase in size of the fluorescently labeled molecular complex upon binding. We use *E. coli* cell-free lysates to rapidly synthesize proteins directly in our sample wells during FCS observation, bypassing the need for laborious cell-line generation and protein purification^7^ (Figure 1). Previously, it was shown that FCS can be performed on proteins produced in cell-free lysates.^8−10^ Here, we also demonstrate the ability to obtain a full binding curve by tracking fluorescent protein expression directly within the cell-free reaction. By combining cell-free protein synthesis and FCS, we simultaneously perform the synthesis and affinity measurement steps, thus enabling a simpler and faster workflow for screening applications.

### System Overview

Our method uses FCS to measure production and binding of fluorescently labeled proteins in cell-free lysates in a one-pot reaction without the need for protein purification (Figure 1). Each reaction required only ∼6 μL, consisting of the cell-free lysate, one purified protein of interest (“Binding partner”), and a plasmid encoding a fluorescently tagged (e.g., green fluorescent protein, GFP) second protein of interest (“Protein2-GFP”) (Figure 1A). Each reaction solution is incubated at room temperature as a thin layer in a sample well between two coverslips, keeping a layer of air above. The layer of air ensures spatially uniform production and detection of the GFP signal across the well over time since oxygen is required for both protein synthesis^11,12^ and GFP maturation.^10,13^ Over the course of protein synthesis, the concentration of Protein2-GFP increases while that of the Binding partner remains constant, so a series of measurements over time produces different concentrations as required for a binding curve.

The fluorescence signal in each sample well is measured over time by FCS (Figure 1B). A 488 nm laser excites the GFP, and emitted photons are selected by a confocal pinhole and detected by two avalanche photodiodes. Over several hours of protein synthesis, an automated stage cycles through each sample well to collect 30 s segments of data consisting of photon arrival times at each detector (Figure 1C). The *g*^(2)^ correlation is calculated as the cross-correlation of the two photon streams to mitigate the nano- to microsecond scale peak due to detector afterpulsing.^14^ As Protein2-GFP is generated over time, the correlation amplitude decreases and the concentration can be determined from the amplitude given the calibrated focal volume. Importantly, the overall diffusion time is initially high since the small amount of Protein2-GFP would be bound to its Binding partner but decreases over time as free Protein2-GFP is produced in excess of the Binding partner. A two-species fit to correlations yields the fraction of Protein2-GFP bound, which, together with its measured concentration, produces a binding curve from which the binding affinity (*K*_D_) is determined.

To demonstrate the feasibility of our method and to investigate its requirements and limitations, we studied the binding of GFP to an anti-GFP antibody. As antibodies can have very high affinities (low *K*_D_), this choice of model proteins enabled us to determine the sensitivity or lower detection limit of the method. We first show that FCS can measure the fluorescence signal in the background of a cell-free lysate and thus track the production of GFP over time without the need for purification. In control experiments, we characterized the binding affinity in buffer and lysate using the conventional method of titrating concentration and determined a low-picomolar *K*_D_ for the antibody. We next performed a time series measurement of the production of GFP in cell-free lysate in the presence of the antibody, thus successfully applying our method to obtain the *K*_D_. Finally, we demonstrate that these measurements can be performed in different sample chamber setups, specifically inside microcapsules, thus potentially substantially increasing the throughput.

## Experimental Section

### Cell Free Protein Synthesis Sample Preparation

GFP was expressed in two *E. coli* derived coupled transcription/translation cell-free protein synthesis lysates, one commercial (Biotech Rabbit BR1400201, hereafter “Rb”) and one homemade (“CC”) (see Supporting Information). Each reaction used specified amounts of an anti-GFP antibody (Abcam 1218) and a pET-15b plasmid encoding His-Avi-tagged GFP. For FCS measurements, sample slides consisted of a 1.8 mm thickness imaging spacer with 3 mm diameter wells (Grace BioLabs no. 666208) containing ∼6 μL of the cell free reaction sandwiched by two glass coverslips. In this way, the bottom half of the well was covered by the solution while leaving a layer of air above to provide adequate oxygen access for cell-free reactions and GFP maturation (Figure S1; see Supporting Information). For microencapsulation, reactions were loaded into glass syringes and kept on ice before connecting to syringe pumps and a home-built double emulsion microfluidic device. See additional details in Supporting Information.

### FCS Data Analysis

We used the 488 nm laser and two green-channel single-photon detectors of a home-built FCS instrument to measure GFP fluorescence signal (see details of instrumentation in Supporting Information). *g*^(2)^ correlations were calculated^15^ using cross correlation between the two green detectors to avoid afterpulsing artifacts, then fitted to one- or two-species models to obtain amplitudes and diffusion times. The simple one-species 2D diffusion model is given by
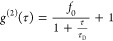
1where *f*_0_ is the fitted amplitude and τ_D_ is the fitted diffusion time. For a time series acquisition during cell free production, the measured concentration *C* at every time point was calculated from the fitted amplitude given the calibrated focal volume *V* and corrected for background contribution using a correction factor β:
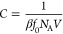
2
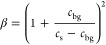
3where *N*_A_ is Avogadro’s number, *c*_bg_ is the background photon count rate measured by the lysate-only sample at the corresponding time point, and *c*_s_ is the count rate of the sample.^16^

The 2-species model is given by
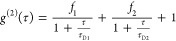
4where *f*_1_ and *f*_2_ correspond to the amplitudes in each population with diffusion times τ_D1_ (GFP alone) and τ_D2_ (GFP bound to antibody). The fraction of GFP bound was then calculated by
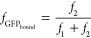
5

For correlation data with two species, we first fitted to the one-species model to confirm the overall trend in the diffusion time. Then the correlation data were fitted to the two-species model with the two diffusion times τ_D1_ (unbound) and τ_D2_ (bound) fixed at values indicated by the one-species fit.

### GFP and Antibody Binding Experiments

In control experiments, we characterized the binding affinity between GFP and its antibody in buffer and lysate by titrating concentration. Since the concentration of the “trace” component was not much lower than the low-picomolar *K*_D_, the data were not fitted to the simple hyperbolic binding equation, and *K*_D_ could not be simply read as the concentration of antibody at *f*_GFP_bound__ = 0.5. Instead, *K*_D_ was determined from a fit to the quadratic binding equation (see Supporting Information for derivation^17^):
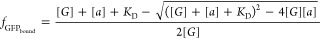
6where [*G*] is the total concentration of GFP, [*a*] is the total concentration of the antibody, and *K*_D_ is the dissociation constant. When [*G*] was held constant and [*a*] titrated, eq 6 was fitted to the measured binding curve (*f*_GFP_bound__ vs [*a*]) with two fitting parameters [*G*] and *K*_D_. When [*a*] was held constant and [*G*] titrated, eq 6 was fitted to *f*_GFP_bound__ vs [*G*] with fitting parameters [*a*] and *K*_D_.

For binding measurements by cell free protein synthesis, samples containing specified concentrations of GFP plasmid and antibody in cell-free lysates were measured over time by FCS. At each time point, the fraction of GFP bound *f*_GFP_bound__ was determined by a two-species fit (eq 5), and the concentration of GFP [*G*] was determined from the fitted amplitude (eqs 2 and 3). The resulting binding data was fitted to eq 6 to obtain the *K*_D_.

## Results

### FCS Tracks Real-Time Production of GFP in Cell-Free Lysates

**Figure 2:**
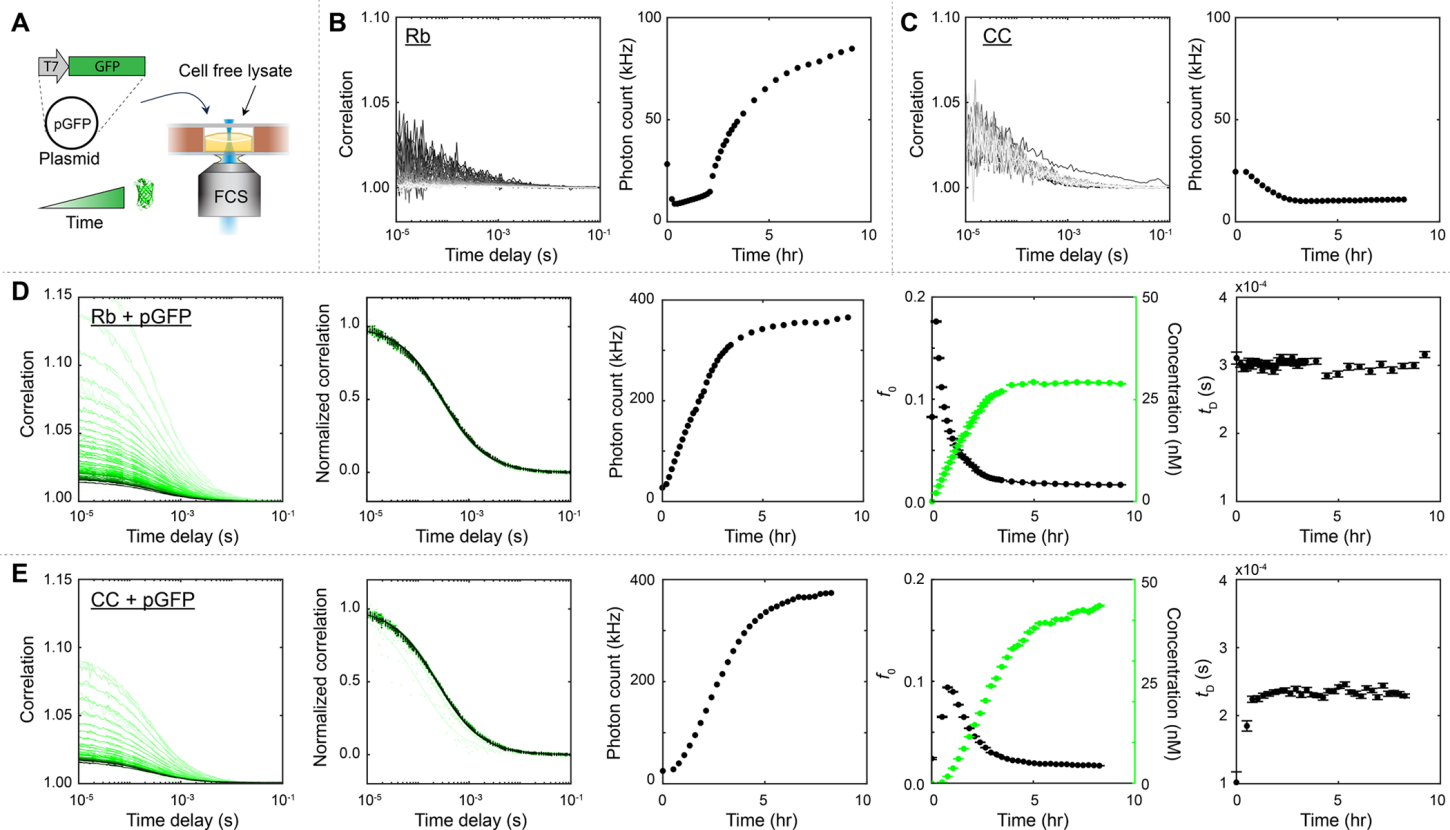
FCS tracks fluorescent proteins synthesized in cell-free lysates in real time. (A) Production of GFP by cell-free reaction containing a GFP plasmid (pGFP) was measured by FCS over time. (B, C) Measurements of commercially available (Rabbit Biotech, Rb) (B) and homemade (CC) (C) lysates without plasmid. Left: correlation curves are colored black (earlier time points) to white (later) and show noise from the lysate background. Right: photon counts for both lysates. (D, E) Measurements of GFP production over time in Rb (D) and CC (E) containing 1 μg/mL (0.23 nM) plasmid at room temperature. From left to right: correlation curves, both raw and normalized, colored light (earlier) to dark (later) green; photon count rate; fitted amplitude *f*_0_ and diffusion times τ_D_ (eq 1) over hours. Concentrations (green) were calculated from *f*_0_ given the calibrated detection volume and were corrected for background signal using the lysate-only data at every time point (eqs 2 and 3). The initially lower *f*_0_ and *t*_D_ are due to the background noise contribution at low GFP concentrations.

To investigate the ability of FCS to measure fluorescent proteins in a nonpure environment, we measured over several hours the expression of GFP in two *E. coli* derived cell-free lysates, one commercial (Rabbit Biotech, “Rb”) and one homemade (“CC”) (Figure 2). While both lysates alone without plasmid had a signal above that of water or buffer, the signals did not show meaningful correlation (Figure 2B,C). Interestingly, the photon counts from both lysates decreased in the initial couple of hours before steadily increasing in Rb while remaining constant in CC. The initial signal decrease may be due to various factors such as photobleaching of fluorescent species from laser excitation and/or consumption or precipitation of these species during the reaction. As lysates are complex mixtures with active processes, and the commercial Rb lysate would likely have a different formulation than the homemade CC lysate, the subsequent increase in background signal observed in Rb is likely due to the making of particular transcription/translation components which are absent from CC.

Above the lysate background, fluorescence signal from the GFP protein was detectable in the low nanomolar range within 0.5 h of plasmid addition (Figure 2D,E). The *g*^(2)^ correlations as a function of time delay τ were fitted to a simple one-species 2D diffusion model (eq 1) to obtain the amplitude *f*_0_ and diffusion time τ_D_ corresponding to the average time a molecule takes to traverse the focal volume. The measured concentration *C* at every time point was calculated from *f*_0_ (eqs 2 and 3). As GFP was produced in the lysates, photon counts increased, *f*_0_ decreased, measured concentration increased, and τ_D_ remained constant, as expected. The constant diffusion time was also apparent in the normalized plots of the correlations. When FCS measurements were started early enough to capture the earliest time points, the total signal from these early points had a dominant contribution from the lysate background, resulting in noisier correlations with lower amplitudes (Figure 2E). This explains the initial increase in fitted *f*_0_ as the amount of GFP increased above the background, as well as the initial lower fitted τ_D_.

Production of GFP was comparable in the Rb and CC lysates. The experiment shown used 1 μg/mL (0.23 nM) plasmid in both lysates and were performed at room temperature, yielding ∼30–40 nM protein by ∼5 h. Interestingly, GFP diffusion times were consistently higher in Rb (τ_D_ ∼ 0.3 ms) than in CC τ_D_ ∼ 0.2 ms). Since τ_D_ measured in CC lysate was the same as that measured in buffer, the higher τ_D_ in Rb lysate must be due to certain conditions or components of that lysate.

### Binding Affinity between GFP and Antibody by Titration of Either Species

**Figure 3:**
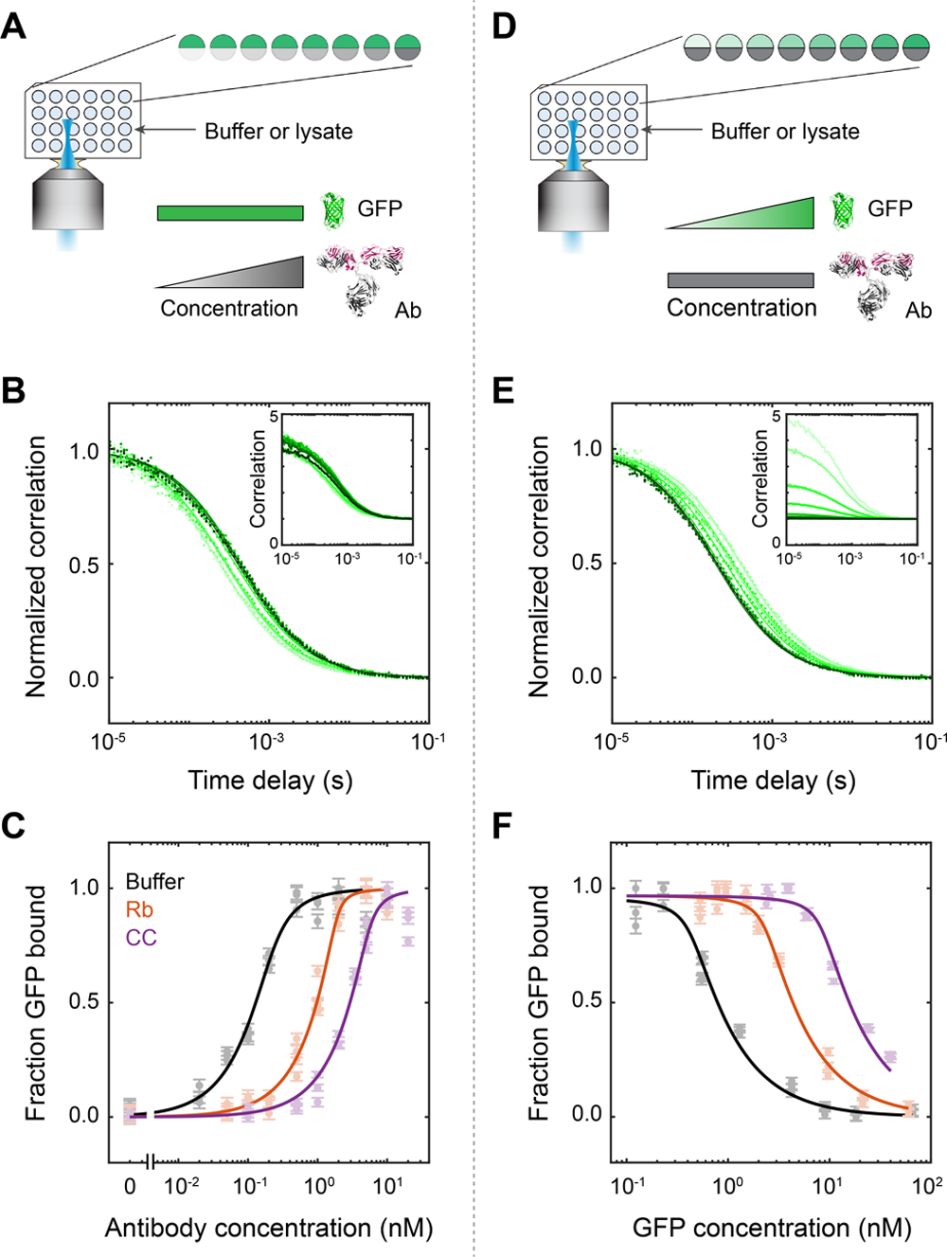
Binding affinity between GFP and an anti-GFP antibody by titration of either species. (A) Samples containing a constant concentration of GFP and varying concentrations of the unlabeled antibody were measured by FCS. (B) Correlation curves shifted right with higher antibody concentration (see Figure S3a for the increase in diffusion times). (C) Binding curves plotted as fraction of GFP bound vs antibody concentration. In buffer, 200 pM GFP was used; fitted *K*_D_ = 0.03 ± 0.01 nM, GFP concentration [*G*] = 0.19 ± 0.03 nM. In Rb lysate, 2 nM GFP was used; fitted *K*_D_ = 0.04 ± 0.02 nM, [*G*] = 1.9 ± 0.1 nM. In CC lysate, 5 nM GFP was used; fitted *K*_D_ = 0.25 ± 0.17 nM, [*G*] = 5.3 ± 0.6 nM. (D) Binding curves were also obtained by varying the GFP concentration while keeping constant the unlabeled antibody concentration. (E) Correlation curves shifted left with higher GFP concentrations (see Figure S3b for the decrease in diffusion times). Inset shows non-normalized curves with lower amplitudes at higher GFP concentrations. (F) Binding curves plotted as fraction of GFP bound vs GFP concentration. In buffer, 400 pM antibody was used; fitted *K*_D_ = 0.02 ± 0.01 nM, antibody concentration [*a*] *=* 0.41 ± 0.02 nM. In Rb lysate, 2 nM antibody was used; fitted *K*_D_ = 0.07 ± 0.02 nM, [*a*] = 2.2 ± 0.1 nM. In CC lysate, 10 nM antibody was used; fitted *K*_D_ = 0.28 ± 0.1 nM, [*a*] = 8.1 ± 0.4 nM.

As control experiments, we first characterized the binding affinity between GFP and its antibody in buffer and lysates using the conventional method of titrating concentration. When the concentration of GFP was held constant and the concentration of antibody was titrated (Figure 3A), correlation curves shifted rightward to higher overall diffusion times when fitted to the one-species model (eq 1) (Figure 3B, Figure S3a). Correlations were then fitted to a two-species model (eq 4) to determine the fraction of GFP bound (eq 5), from which fitting to the quadratic binding equation gave the dissociation constant *K*_D_ and GFP concentration [*G*] (eq 6). In buffer, 200 pM GFP was used, giving fitted *K*_D_ = 0.03 ± 0.01 nM and [*G*] = 0.19 ± 0.03 nM, the latter agreeing well with the known 200 pM GFP used (Figure 3C). In lysates, larger amounts of GFP were required to measure signals above the higher lysate background. In Rb lysate using 2 nM GFP, fitted *K*_D_ = 0.04 ± 0.02 nM and [*G*] = 1.9 ± 0.1 nM (Figure 3C), consistent with the *K*_D_ measured in buffer and the known amount of GFP used. In CC lysate using 5 nM GFP, fitted *K*_D_ = 0.25 ± 0.17 nM and [*G*] = 5.3 ± 0.6 nM. While [*G*] was accurately recapitulated, the measured apparent *K*_D_ was higher in CC lysate than in buffer and had greater uncertainty due in part to the use of GFP at a concentration much greater than *K*_D_.

A binding curve could also be obtained by titrating the concentration of GFP while keeping constant that of the unlabeled antibody (Figure 3D). This case mimics the proposed cell-free expression approach, in which the fluorescent component increases in concentration over time. As GFP increased in concentration in excess of the antibody used, correlation curves shifted leftward to lower overall diffusion times since FCS tracks the GFP (Figure 3E, Figure S3b). Equation 6 is fitted to the fraction of GFP bound as a function of GFP concentration. In buffer using 400 pM antibody, we obtained fitted *K*_D_ = 0.02 ± 0.01 nM and antibody concentration [*a*] = 0.41 ± 0.02 nM (Figure 3F), consistent with both the *K*_D_ determined by the standard titration method (Figure 3A–C) and the known amount of antibody used. In Rb lysate using 2 nM antibody, fitted *K*_D_ = 0.06 ± 0.02 nM and [*a*] = 2.2 ± 0.1 nM, and in CC lysate using 10 nM antibody, fitted *K*_D_ = 0.28 ± 0.1 nM and [*a*] = 8.1 ± 0.4 nM (Figure 3F). Again, while [*a*] was accurately recapitulated in both lysates, the measured apparent *K*_D_ was higher in the CC lysate due in part to the higher antibody concentration used which precludes accurate determination of the very low *K*_D_.^18^ In addition, it is also possible that the binding affinity between GFP and the antibody was slightly altered under the conditions of the CC lysate buffer.

In summary, we measured the binding affinity of GFP and its antibody by titrating either GFP or the antibody and found good agreement between the two methods that yielded a picomolar *K*_D_.

### Determination of Binding Affinity from FCS Measurements of Protein Synthesis over Time in Cell-Free Lysates

**Figure 4:**
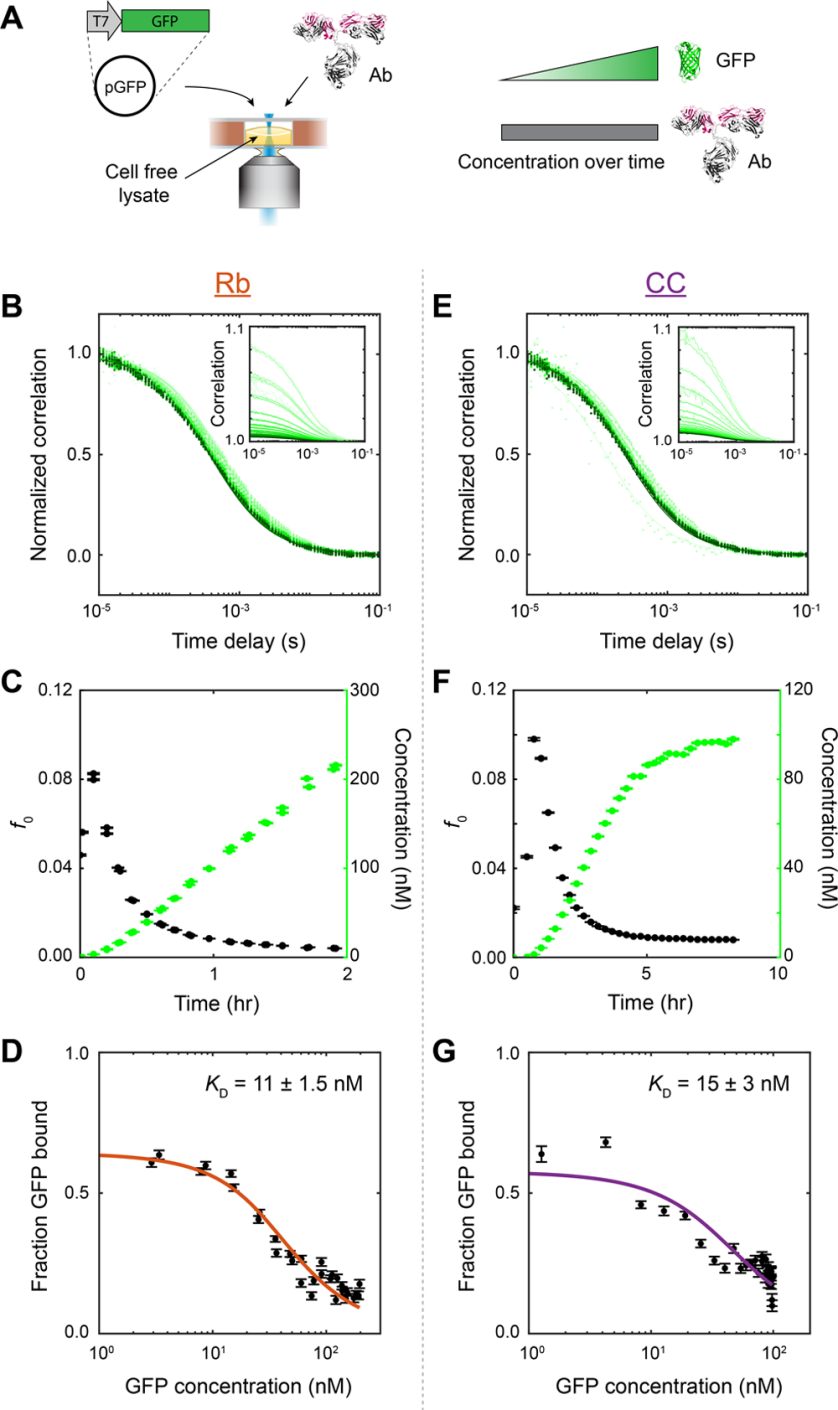
Determination of binding affinity from FCS measurements of protein synthesis over time in cell-free lysates. (A) Samples containing GFP plasmid and antibody in cell-free lysate were measured over time by FCS for production of GFP and antibody binding. (B–D) 5 μg/mL (1.2 nM) plasmid and 20 nM antibody in Rb lysate. Correlation curves (B) shifted left as the GFP concentration increased over time. Total GFP concentrations were determined from the total amplitude *f*_0_ of two-species fits (eq 4) and background subtracted (eqs 2 and 3) using lysate-only data at each time point (C). Fraction of GFP bound calculated from two-species fits (eqs 4 and 5) is plotted against the measured GFP concentrations (D). Fitting the quadratic binding equation (eq 6) produced *K*_D_ = 11 ± 1.5 nM and antibody concentration [*a*] = 19 ± 1.3 nM. (E–G) Correlation curves (E), total amplitudes and background-subtracted concentrations (F), and binding curve (G) for measurements of 2 μg/mL (0.5 nM) plasmid and 20 nM antibody in CC lysate. Fitted *K*_D_ = 15 ± 3 nM, [*a*] = 20 ± 2 nM.

Samples containing GFP plasmid and antibody in both cell-free lysates were measured over time by FCS for the production of GFP and binding to antibody (Figure 4A). We used appropriate plasmid and antibody concentrations to achieve accurate picomolar/low-nanomolar affinity measurements (see Figure S2 and note in Supporting Information).

In a ∼6 μL solution containing 5 μg/mL (1.2 nM) plasmid and 20 nM antibody in Rb lysate, GFP was quickly produced and reached ∼200 nM within 2 h (Figure 4B,C). Further data beyond 2 h were not collected because the amount of GFP reached 10× higher than the antibody concentration and was sufficient for a binding curve. Normalized correlation curves shifted left as free GFP was produced, the concentration of which increased above that of the antibody (Figure 4B). The total GFP concentration at each time point was determined from the total amplitude *f*_0_ = *f*_1_ + *f*_2_ of two-species fits (eq 4) and background subtracted (eqs 2 and 3) using lysate-only data (Figure 4C). The binding curve results from plotting the fraction of GFP bound calculated from two-species fits against the measured GFP concentrations (Figure 4D). Fitting the quadratic binding equation (eq 6) produced *K*_D_ = 11 ± 1.5 nM and [*a*] = 19 ± 1.3 nM. This process was repeated in CC lysate using 2 μg/mL (0.5 nM) plasmid and 20 nM antibody and yielded *K*_D_ = 15 ± 3 nM and [*a*] = 20 ± 2 nM (Figure 4E–G). In both lysates, the fitted antibody concentration [*a*] matched the known concentration used (20 nM), while the *K*_D_ ∼ 10 nM was much higher than the picomolar affinity determined in control experiments (Figure 3).

The higher measured *K*_D_ values resulted from the relatively high antibody concentration used and from the reasons discussed in the next section. The high antibody concentration was necessary for measurements above the lysate background (see Supporting Information) but precluded accurate determination of the low picomolar *K*_D_. These higher measured *K*_D_ values therefore establish an upper bound on the true binding affinity. The ability to measure a low-nanomolar *K*_D_ is still very informative because weak and strong binders would be distinguishable in a large initial screen and because most biological affinities range from nanomolar to micromolar. Importantly, we have demonstrated that this determination of *K*_D_ is possible within a couple of hours of starting a one-pot cell-free reaction.

### Experimental Factors Affecting Determination of Binding Affinity

It is known that accurate determination of binding affinity is not possible in the presence of experimental noise when the concentration of the “trace species” is high compared to the *K*_D_, leading to a measured value greater than the true *K*_D_ ^18^ (Figure S4). To test that this explains the much higher apparent *K*_D_ (∼10 nM) measured by tracking real-time protein synthesis (Figure 4) in comparison to the true *K*_D_ (∼30 pM) (Figure 3), and to characterize the variability in the method, we performed multiple cell-free production experiments using different concentrations of the trace species (antibody) ranging from [*a*] ∼ 3 nM to 80 nM. Note that the lowest [*a*] used was still much higher than the picomolar *K*_D_ because of the constraint due to lysate background: a much lower picomolar [*a*] would have meant that by the time the GFP signal was measurable above the lysate background, at a few nanomolar GFP, almost all of the GFP would have been free, as is the case for the remainder of the time series, thus yielding an uninformative null binding curve.

**Figure 5:**
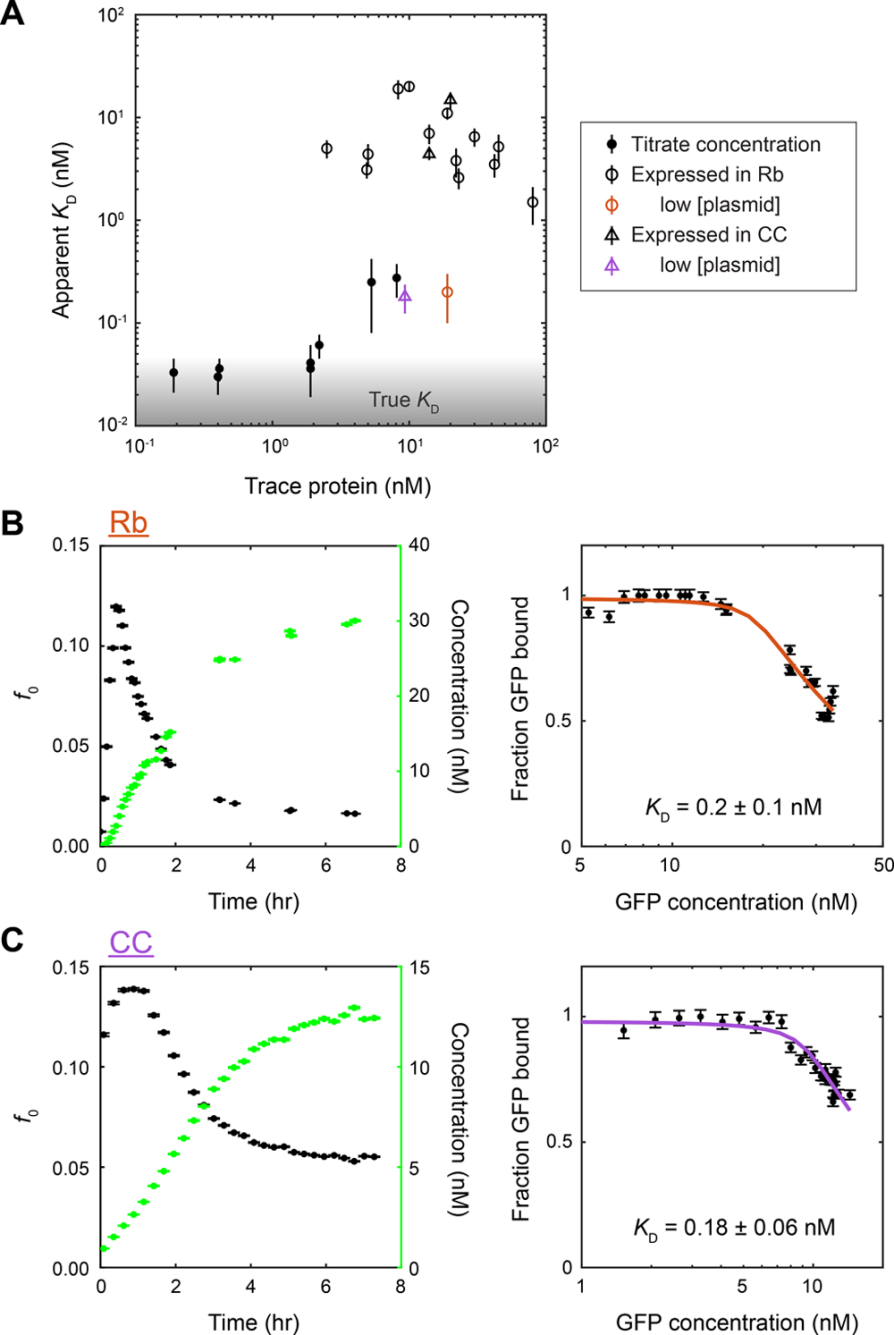
Use of higher concentrations of trace binding partner and other experimental factors establish upper bound on the *K*_D_ measured by cell-free protein synthesis. (A) Compilation of the measured *K*_D_ vs the “trace protein” concentration *P* used from all binding experiments. Accurate determination of *K*_D_ typically requires *P* < *K*_D_, a condition often not met in biological experiments due to practical constraints. Control experiments, in which subnanomolar *P* (antibody or GFP concentration) were used (solid markers) (from Figure 3), suggested that the true affinity was ∼30 pM or less (shaded area). Much higher *K*_D_ values were measured from real-time protein synthesis experiments (open markers) due to a combination of the much higher *P* (antibody concentration) required in the lysate background, GFP maturation time, and/or nonequilibrium binding. Slowing down and lowering protein expression produced *K*_D_ measurements more consistent with control experiments (colored open markers) (B, C). (B) Binding experiment from protein synthesis using 0.7 μg/mL (0.17 nM) GFP plasmid and 20 nM antibody in Rb lysate, analyzed as in Figure 4. Fitted *K*_D_ = 0.2 ± 0.1 nM, antibody concentration [*a*] = 18.7 ± 0.5 nM (orange open circle in (A)). (C) Binding experiment using 0.5 μg/mL (0.12 nM) plasmid and 20 nM antibody in CC lysate. Fitted *K*_D_ = 0.18 ± 0.06 nM, antibody concentration [*a*] = 9.3 ± 0.2 nM (purple open triangle in (A)).

We found that the measured *K*_D_ reproducibly ranged between 1 and 20 nM across all experiments (open black markers in Figure 5A). While these values were ∼100–500× higher than the picomolar *K*_D_ values measured in control experiments performed without real-time protein production (solid black markers in Figure 5A), they provided a confident low-nanomolar upper bound. However, the measured *K*_D_ values were independent of the antibody concentration. At [*a*] ∼ 10 nM and below, *K*_D_ measured by protein synthesis was still ∼10–50× higher than those measured by control experiments at similar [*a*]. Thus, the use of [*a*] ≫ *K*_D_ could not be the only factor causing the higher measured *K*_D_.

Two additional factors may be the maturation time of GFP and nonequilibrium binding. First, maturation times of fluorescent proteins have been found to range from several minutes to upward of an hour,^19^ before which the protein is folded but nonfluorescent. During this time in a protein synthesis experiment, dark GFP molecules would have been able to bind the antibody but would not have been detected by FCS, thus sequestering a fraction of the antibody to be unavailable to bind to the matured bright GFP molecules. This would result in a lower apparent fraction of GFP bound, as measured by bright GFP molecules, and thus a higher apparent *K*_D_. Second, proteins that have very low *K*_D_ may have very slow binding on rates.^18^ In our experiments that measured binding during 2–5 h of GFP synthesis, the production and detection of GFP might have outpaced the on rate or time required for equilibrium binding, resulting again in a lower apparent fraction of GFP bound and higher apparent *K*_D_.

To test both factors, we slowed the rate of protein synthesis by using a lower GFP plasmid concentration in binding experiments. GFP production rate was ∼7 nM/h in Rb lysate using 0.7 μg/mL plasmid (Figure 5B) and ∼2.5 nM/h in CC lysate using 0.5 μg/mL (Figure 5C), compared to ∼100 nM/h and 20 nM/h using 5 μg/mL and 2 μg/mL previously in the two lysates, respectively (Figure 4). We found that indeed the measured *K*_D_ was lowered: *K*_D_ = 0.2 ± 0.1 nM in Rb lysate, *K*_D_ = 0.18 ± 0.06 nM in CC lysate (Figure 5B,C). By slowing down protein expression, these *K*_D_ measurements were now consistent with control experiments done at similar antibody concentrations (colored open markers vs black solid markers in Figure 5A). Furthermore, in the presence of experimental noise, these binding curves corresponding to ∼200 pM *K*_D_ are indistinguishable from those corresponding to ∼30 pM *K*_D_ when using excess (20 nM) antibody (Figure S4), which suggests that now the *K*_D_ determination is limited by the high trace protein concentration.

### Determination of Binding Affinity from Protein Synthesis Inside Microcapsules

**Figure 6:**
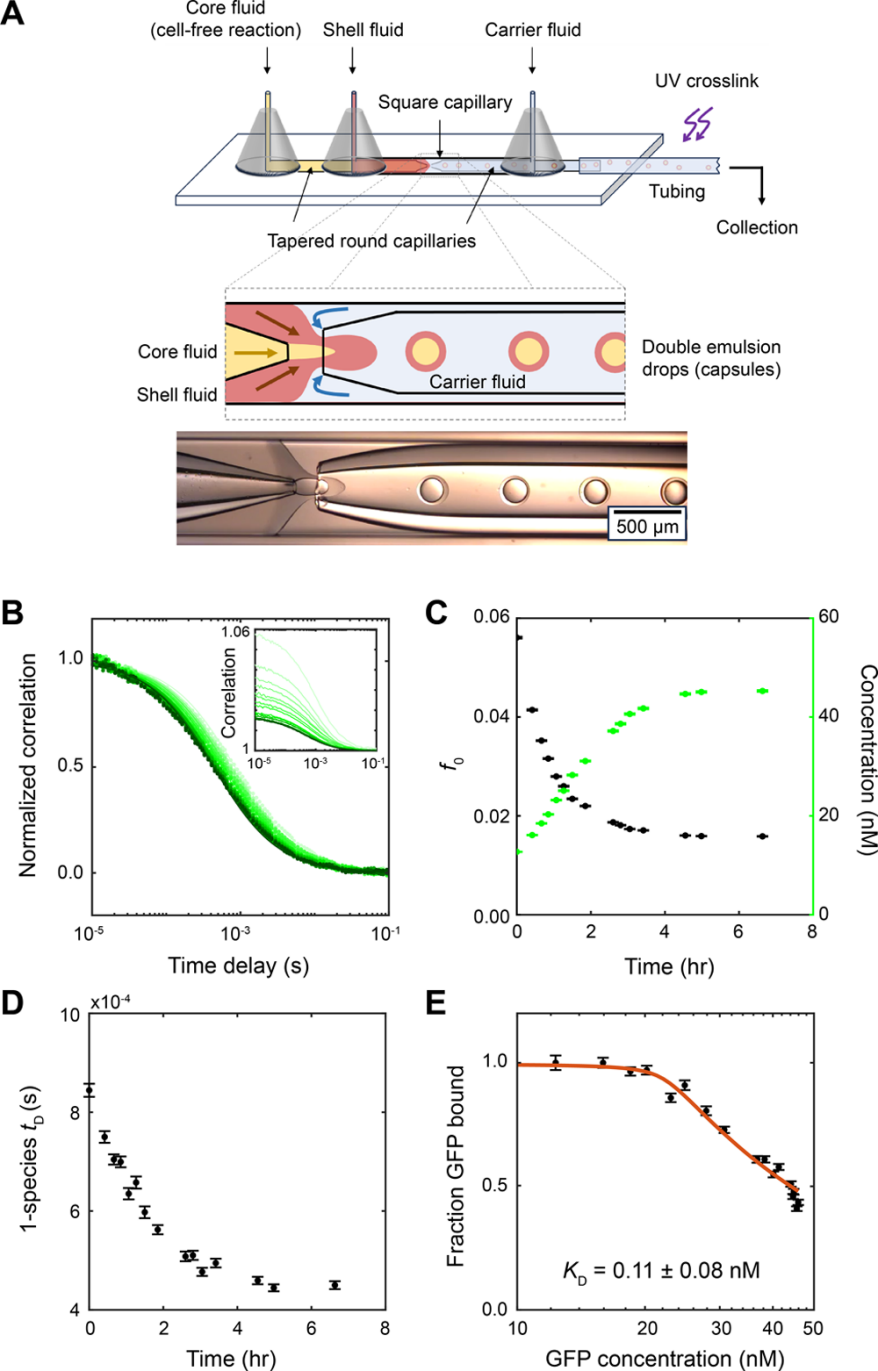
Determination of binding affinity from protein synthesis inside microcapsules. (A) Top: schematic of double emulsion droplet generation on a microfluidic device. Through the flow of fluids in two round capillaries with tapered openings within a larger square capillary, the core fluid (cell-free reaction) (yellow) is packaged into droplets with a polymer shell (red) surrounded by the carrier fluid (light blue). Each capsule contains ∼10 nL or less of reaction. Droplets pass under a UV lamp to cross-link the shell polymer before collection and measurement of the capsules by FCS. Bottom: image of droplet formation in the region of the interface between the three fluids. (B–E) FCS measurements of protein synthesis and binding inside a microcapsule containing 1.45 μg/mL (0.34 nM) GFP plasmid and 20 nM antibody in Rb lysate. (B) Correlation curves shifted left as the GFP concentration increased over time. (C) Total background-subtracted GFP concentrations determined from the total correlation amplitude *f*_0_. (D) Overall diffusion times decreased over time as the GFP concentration increased. (E) Fraction of GFP bound calculated from two-species fits (eqs 4 and 5) plotted against the measured GFP concentrations. Fitting the quadratic equation (eq 6) gave *K*_D_ = 0.11 ± 0.08 nM and antibody concentration [*a*] = 22.2 ± 0.5 nM.

Toward high throughput screening applications, our FCS-based in-lysate binding method may be adapted to perform measurements in various sample formats including well plates, microfluidic chambers, and microcapsules. Microencapsulation provides a method to compartmentalize thousands to millions of independent reactions and enables the use of digital microfluidics to achieve unprecedented statistics, with benefits widely demonstrated in the literature, for example, by the development of real-time digital droplet PCR.^20−22^ Through a double emulsion droplet generation process using a homemade microfluidic device, we packaged cell-free reactions into 200–300 μm droplets enclosed by a polymer shell, which are then stabilized by UV cross-linking of the polymer (Figure 6A). Each capsule contains only ∼10 nL of solution. It has not been shown in the literature whether a cell-free reaction can proceed inside microcapsules or whether the fluorescence correlation signal can be measured through the capsule shell.

We made microcapsules containing cell-free lysate, GFP plasmid, and GFP antibody, placed the microcapsules on a glass coverslip, and measured the FCS signal inside for several hours. We were able to measure both protein synthesis and binding, consistent with previous measurements made in wells. Inside a capsule containing 1.45 μg/mL (0.34 nM) GFP plasmid and 20 nM antibody in Rb lysate, GFP concentration increased to ∼45 nM in ∼4 h, while overall diffusion times decreased (Figure 6B–D). A fit of the quadratic binding equation (eq 6) to the binding curve gave *K*_D_ = 0.11 ± 0.08 nM and antibody concentration [*a*] = 22.2 ± 0.5 nM (Figure 6E), both agreeing with the previous binding measurements and known amounts of antibody used. The adaptability of our method to microcapsules is a key demonstration of the feasibility of using this approach to high throughput affinity screening.

## Discussion

Relying on cell-free protein synthesis has both limitations and advantages. A fundamental requirement is that the protein must be amenable to expression, and to a high enough extent, in one of the ∼10–20 existing cell-free platforms.^12^ But this may also be a challenge in cell-based systems for difficult-to-synthesize proteins. In fact, some proteins that do not express well in cells due to toxicity or aggregation may be better tolerated and produced in cell-free systems. For example, soluble membrane proteins may be made co-translationally in lipid nanodiscs by cell-free systems.^23−25^ Furthermore, for proteins that do not purify well, the ability to measure binding in the lysate environment bypasses the purification requirement. Finally, tracking protein synthesis by FCS provides a wealth of information in addition to binding affinity: expression levels, rates, and aggregation tendencies, all valuable information for screening new protein variants. All of this information is obtained with minimal amounts of cell-free reaction: ∼6 μL for reactions in wells and less than 10 nL inside a microcapsule.

Although measuring binding simultaneous with protein synthesis eliminates the need for protein purification and significantly speeds up affinity assessment, this strategy has inherent limitations. First, without purification, the lowest detectable limit of the expressed protein is ∼1 nM in order to be above the lysate background. Second, binding may not reach equilibrium since measurements are made during the continuing protein synthesis. Third, the fluorescent protein used to label the protein of interest has a non-negligible maturation time, which obscures the readout of the true protein concentration. In this work, the choice of using GFP and its antibody as the model pair, with their low-picomolar *K*_D_, enabled us to characterize these limitations. We found that these three factors limit the lowest measurable *K*_D_ to the low nanomolar range, a sensitivity sufficient for many screening applications, nonetheless.

We found that these limitations may be mitigated by slowing the rate of protein synthesis, allowing the measurement of subnanomolar *K*_D_ (Figure 5). The rate can be controlled by adjusting the plasmid concentration and/or temperature. To mitigate complications arising from GFP maturation time, we kept a layer of air above the thin layer of cell-free solution. Future improvements may include saturating the reaction with oxygen or supplying higher oxygen-content air above the solution. An alternative strategy may employ a split-GFP technique in which a prematured GFP1–10 is added to the cell-free reaction to complement the protein-GFP11 being expressed, since prematuration has been shown to significantly speed up signal detection.^26^ Finally, to avoid the issue of fluorescent protein maturation entirely, chemical dyes may be used as the fluorescent label instead of fluorescent proteins. Specifically, BODIPY-FL-labeled lysine (Promega Corp.) may be incorporated into the protein during translation, and the free lysine-dye component in the correlation data can be removed during analysis.

While a shift in diffusion time of the FCS correlation curves provides a simple readout of binding requiring a simple one-color FCS setup, the diffusion times of the two species (unbound and bound) should differ by at least 1.6× to be distinguishable under typical conditions.^27^ This requirement means that the masses of the two species must differ by at least 4×, since diffusion time is inversely proportional to the radius (cubed root of mass). In the current work, FCS easily and reliably measured the increase in diffusion time of GFP upon antibody binding based on ∼6× increase in mass, from ∼30 kDa GFP to ∼30 + 150 kDa GFP + antibody. Measurements of other binding pairs that have smaller changes in size can be made possible using dual-focus FCS, which can measure angstrom-scale changes,^28,29^ or dual-color FCS, in which binding is indicated by cross-correlation between proteins labeled with different dyes.^30,31^

## Conclusion

As both core components (cell-free protein synthesis, FCS) of the method presented in this article are well established and relatively available, our approach represents a simple yet novel integration with widespread applicability. Its lack of need for complex instrumentation or biological systems grants accessibility while inviting advanced technological development.

By combining cell-free protein synthesis and FCS, we simultaneously perform the synthesis and affinity measurement steps, thus enabling a simpler, faster workflow for screening. Suppose we need to measure whether variants of a protein bind to another protein of interest, for example, in a screen for antibody fragments targeting the SARS-CoV-2 spike protein. Conventionally, each variant must be separately expressed in cells and purified. Then, for each variant, multiple samples containing different concentrations of the protein must be prepared and measured to obtain a binding curve. This process typically takes several days, from protein expression to purification to measurements. In contrast, our approach measures production and binding in a one-pot reaction for each variant without the need for purification or prescription of different concentrations, using only a few hours of cell-free protein synthesis (Figure S5). This significantly simplifies and speeds up measurements, enabling scalability and high throughput in screens for biomolecular binding.

We have demonstrated the proof of concept of performing simultaneous measurements of protein production and binding, leaving the potential for extensive future technology development for high throughput applications. In a high throughput screen, repeated measurements are to be made over time on multiple samples to track protein production and binding (Figure S5). Currently each measurement takes 30 s on the conventional FCS, which enables thousands of measurements in a day. By using multispot FCS^32^ or selective plane illumination FCS (SPIM-FCS)^33^ coupled to single photon avalanche diode (SPAD) array detectors,^34^ tens to thousands of measurements can be made in parallel, thus reducing measurement times to <1 s and enabling potentially many thousands of samples to be measured in 1 day. This ability presents a truly revolutionary potential in high throughput studies of intermolecular interactions across the biosciences and medicine.

## Supporting Information Available

Additional detailed experimental methods, results of supporting experiments, and supporting figures (PDF)

## References

[ref1] Macarron R.; Banks M. N.; Bojanic D.; Burns D. J.; Cirovic D. A.; Garyantes T.; Green D. V.; Hertzberg R. P.; Janzen W. P.; Paslay J. W.; Schopfer U.; Sittampalam G. S. Impact of high-throughput screening in biomedical research. Nat. Rev. Drug Discov 2011, 10 (3), 188–195. 10.1038/nrd3368. 21358738

[ref2] Hummer A. M.; Abanades B.; Deane C. M. Advances in computational structure-based antibody design. Curr. Opin Struct Biol. 2022, 74, 102379 10.1016/j.sbi.2022.102379. 35490649

[ref3] Zhu F.; Bourguet F. A.; Bennett W. F. D.; Lau E. Y.; Arrildt K. T.; Segelke B. W.; Zemla A. T.; Desautels T. A.; Faissol D. M. Large-scale application of free energy perturbation calculations for antibody design. Sci. Rep 2022, 12 (1), 12489 10.1038/s41598-022-14443-z. 35864134 PMC9302960

[ref4] Schwille P.; Haustein E. Fluorescence Correlation Spectroscopy. An Introduction to Its Concepts and Applications; Biophysical Society, 2002.

[ref5] Magde D.; Elson E.; Webb W. W. Thermodynamic Fluctuations in a Reacting System---Measurement by Fluorescence Correlation Spectroscopy. Phys. Rev. Lett. 1972, 29 (11), 705–708. 10.1103/PhysRevLett.29.705.

[ref6] Rigler R.; Mets Ü.; Widengren J.; Kask P. Fluorescence correlation spectroscopy with high count rate and low background: analysis of translational diffusion. Eur. Biophys. J. 1993, 22, 169–175. 10.1007/BF00185777.

[ref7] Hunt A. C.; Vogeli B.; Hassan A. O.; Guerrero L.; Kightlinger W.; Yoesep D. J.; Kruger A.; DeWinter M.; Diamond M. S.; Karim A. S.; Jewett M. C. A rapid cell-free expression and screening platform for antibody discovery. Nat. Commun. 2023, 14 (1), 3897 10.1038/s41467-023-38965-w. 37400446 PMC10318062

[ref8] Mureev S.; Kovtun O.; Nguyen U. T. T.; Alexandrov K. Species-independent translational leaders facilitate cell-free expression. Nat. Biotechnol. 2009, 27 (8), 747–752. 10.1038/nbt.1556. 19648909

[ref9] Sierecki E.; Giles N.; Polinkovsky M.; Moustaqil M.; Alexandrov K.; Gambin Y. A cell-free approach to accelerate the study of protein–protein interactions in vitro. Interface Focus 2013, 3 (5), 20130018 10.1098/rsfs.2013.0018. 24511386 PMC3915825

[ref10] Macdonald P. J.; Chen Y.; Mueller J. D. Chromophore maturation and fluorescence fluctuation spectroscopy of fluorescent proteins in a cell-free expression system. Anal. Biochem. 2012, 421 (1), 291–298. 10.1016/j.ab.2011.10.040. 22093611 PMC3367886

[ref11] Lin X.; Zhou C.; Zhu S.; Deng H.; Zhang J.; Lu Y. O2-Tuned Protein Synthesis Machinery in Escherichia coli-Based Cell-Free System. Front. Bioeng. Biotechnol. 2020, 8, 312 10.3389/fbioe.2020.00312. 32328487 PMC7160232

[ref12] Gregorio N. E.; Levine M. Z.; Oza J. P. A User’s Guide to Cell-Free Protein Synthesis. Methods and Protocols 2019, 2 (1), 24 10.3390/mps2010024. 31164605 PMC6481089

[ref13] Heim R.; Prasher D. C.; Tsien R. Y. Wavelength Mutations and Posttranslational Autoxidation of Green Fluorescent Protein. Proc. Natl. Acad. Sci. U. S. A. 1994, 91 (26), 12501–12504. 10.1073/pnas.91.26.12501. 7809066 PMC45466

[ref14] Widengren J.; Mets U.; Rigler R. Fluorescence correlation spectroscopy of triplet states in solution: a theoretical and experimental study. J. Phys. Chem. 1995, 99 (36), 13368–13379. 10.1021/j100036a009.

[ref15] Laurence T. A.; Fore S.; Huser T. Fast, flexible algorithm for calculating photon correlations. Opt. Lett. 2006, 31 (6), 829–831. 10.1364/OL.31.000829. 16544638

[ref16] Buschmann V.; Krämer B.; Koberling F.; Macdonald R. M., Ruttinger S.. Quantitative FCS: Determination of the Confocal Vol. by FCS and Bead Scanning with the MicroTime 200. PicoQuant GmbH, 2009.

[ref17] Segel I. H. Enzyme Kinetics; Behavior and Analysis of Rapid Equilibrium and Steady-State Enzyme Systems; Wiley, 1975.

[ref18] Jarmoskaite I.; AlSadhan I.; Vaidyanathan P. P.; Herschlag D. How to measure and evaluate binding affinities. eLife 2020, 9, e57264 10.7554/eLife.57264. 32758356 PMC7452723

[ref19] Balleza E.; Kim J. M.; Cluzel P. Systematic characterization of maturation time of fluorescent proteins in living cells. Nat. Methods 2018, 15 (1), 47–51. 10.1038/nmeth.4509. 29320486 PMC5765880

[ref20] Kiss M. M.; Ortoleva-Donnelly L.; Beer N. R.; Warner J.; Bailey C. G.; Colston B. W.; Rothberg J. M.; Link D. R.; Leamon J. H. High-Throughput Quantitative Polymerase Chain Reaction in Picoliter Droplets. Anal. Chem. 2008, 80 (23), 8975–8981. 10.1021/ac801276c. 19551929 PMC2771884

[ref21] Beer N. R.; Wheeler E. K.; Lee-Houghton L.; Watkins N.; Nasarabadi S.; Hebert N.; Leung P.; Arnold D. W.; Bailey C. G.; Colston B. W. On-Chip Single-Copy Real-Time Reverse-Transcription PCR in Isolated Picoliter Droplets. Anal. Chem. 2008, 80 (6), 1854–1858. 10.1021/ac800048k. 18278951

[ref22] Beer N. R.; Hindson B. J.; Wheeler E. K.; Hall S. B.; Rose K. A.; Kennedy I. M.; Colston B. W. On-Chip, Real-Time, Single-Copy Polymerase Chain Reaction in Picoliter Droplets. Anal. Chem. 2007, 79 (22), 8471–8475. 10.1021/ac701809w. 17929880

[ref23] Cappuccio J. A.; Blanchette C. D.; Sulchek T. A.; Arroyo E. S.; Kralj J. M.; Hinz A. K.; Kuhn E. A.; Chromy B. A.; Segelke B. W.; Rothschild K. J.; Fletcher J. E.; Katzen F.; Peterson T. C.; Kudlicki W. A.; Bench G.; Hoeprich P. D.; Coleman M. A. Cell-free co-expression of functional membrane proteins and apolipoprotein, forming soluble nanolipoprotein particles. Mol. Cell Proteomics 2008, 7 (11), 2246–2253. 10.1074/mcp.M800191-MCP200. 18603642 PMC2577204

[ref24] Katzen F.; Fletcher J. E.; Yang J. P.; Kang D.; Peterson T. C.; Cappuccio J. A.; Blanchette C. D.; Sulchek T.; Chromy B. A.; Hoeprich P. D.; Coleman M. A.; Kudlicki W. Insertion of membrane proteins into discoidal membranes using a cell-free protein expression approach. J. Proteome Res. 2008, 7 (8), 3535–3542. 10.1021/pr800265f. 18557639

[ref25] Ly S.; Bourguet F.; Fischer N. O.; Lau E. Y.; Coleman M. A.; Laurence T. A. Quantifying Interactions of a Membrane Protein Embedded in a Lipid Nanodisc using Fluorescence Correlation Spectroscopy. Biophys. J. 2014, 106 (2), L05–L08. 10.1016/j.bpj.2013.12.014. 24461026 PMC3907250

[ref26] Lundqvist M.; Thalén N.; Volk A.-L.; Hansen H. G.; von Otter E.; Nygren P.-Å.; Uhlen M.; Rockberg J. Chromophore pre-maturation for improved speed and sensitivity of split-GFP monitoring of protein secretion. Sci. Rep. 2019, 9 (1), 310 10.1038/s41598-018-36559-x. 30670736 PMC6342966

[ref27] Meseth U.; Wohland T.; Rigler R.; Vogel H. Resolution of Fluorescence Correlation Measurements. Biophys. J. 1999, 76 (3), 1619–1631. 10.1016/S0006-3495(99)77321-2. 10049342 PMC1300138

[ref28] Dertinger T.; Pacheco V.; von der Hocht I.; Hartmann R.; Gregor I.; Enderlein J. Two-focus fluorescence correlation spectroscopy: a new tool for accurate and absolute diffusion measurements. ChemPhysChem 2007, 8 (3), 433–443. 10.1002/cphc.200600638. 17269116

[ref29] Dertinger T.; Loman A.; Ewers B.; Muller C. B.; Kramer B.; Enderlein J. The optics and performance of dual-focus fluorescence correlation spectroscopy. Opt Express 2008, 16 (19), 14353–14368. 10.1364/OE.16.014353. 18794971

[ref30] Miller A. E.; Hollars C. W.; Lane S. M.; Laurence T. A. Fluorescence Cross-Correlation Spectroscopy as a Universal Method for Protein Detection with Low False Positives. Anal. Chem. 2009, 81 (14), 5614–5622. 10.1021/ac9001645. 19522509

[ref31] Schwille P.; Meyer-Almes F. J.; Rigler R. Dual-color fluorescence cross-correlation spectroscopy for multicomponent diffusional analysis in solution. Biophys. J. 1997, 72 (4), 1878–1886. 10.1016/S0006-3495(97)78833-7. 9083691 PMC1184381

[ref32] Ingargiola A.; Segal M.; Gulinatti A.; Rech I.; Labanca I.; Maccagnani P.; Ghioni M.; Weiss S.; Michalet X. 48-spot single-molecule FRET setup with periodic acceptor excitation. J. Chem. Phys. 2018, 148 (12), 123304 10.1063/1.5000742. 29604810 PMC5669981

[ref33] Wohland T.; Shi X.; Sankaran J.; Stelzer E. H. Single plane illumination fluorescence correlation spectroscopy (SPIM-FCS) probes inhomogeneous three-dimensional environments. Opt Express 2010, 18 (10), 10627–10641. 10.1364/OE.18.010627. 20588915

[ref34] Singh A. P.; Krieger J. W.; Buchholz J.; Charbon E.; Langowski J.; Wohland T. The performance of 2D array detectors for light sheet based fluorescence correlation spectroscopy. Opt Express 2013, 21 (7), 8652–8668. 10.1364/OE.21.008652. 23571955

