# Supplementary material for: Real-Time Affinity Measurements of Proteins Synthesized in Cell-Free Lysate Using Fluorescence Correlation Spectroscopy
Source: Anal Chem. 2025 Apr 30;97(18):9638–47. doi: 10.1021/acs.analchem.4c05485 (PMC12080479; doi:10.1021/acs.analchem.4c05485)
Supplement: Supplementary file 1 — ac4c05485_si_001.pdf [file ac4c05485_si_001.pdf]

Supporting Information for

Real-time affinity measurements of proteins synthesized in cell-free lysate using fluorescence correlation spectroscopy

Chao Liu[1], Steven A. Hoang-Phou[1], Congwang Ye[2], Emma J. Laurence[1], Matthew J. Laurence[1], Erika J. Fong[2], Nikki M. Hammond[1], B. Dillon Vannest[1], Nicholas N. Watkins[2], Ted A. Laurence[3], Matthew A. Coleman[1,*]

[1] Biosciences and Biotechnology Division, Lawrence Livermore National Laboratory, Livermore, CA 94550
[2] Materials Engineering Division, Lawrence Livermore National Laboratory, Livermore, CA 94550
[3] Materials Science Division, Lawrence Livermore National Laboratory, Livermore, CA 94550

* Correspondence: coleman16@llnl.gov

Contents

Detailed experimental methods
Additional supporting results
Figures S1-S5
Supporting Information References

Detailed Methods

Preparation of homemade cell-free protein synthesis lysate ("CC")

Overnight starter cultures of ClearColi ("CC") BL21(DE3) (Biosearch Technologies) grown in 2x YT media supplemented to 1% NaCl at 37 C 225 rpm were diluted 1:50 into multiple 2 L baffled shake flasks containing 500 mL of 2x YT/1% NaCl media. Cultures were grown until OD600 reached 0.5-0.7 and induced with 1 mM IPTG for 4h. Cells were harvested by centrifugation at 8000 rpm in an SLA-3000 rotor, washed with Buffer A (10 mM Tris base, 14 mM magnesium glutamate, 60 mM potassium glutamate, 1 mM DTT), centrifuged again at 6000 rpm, and resuspended with 1 mL of Buffer A per gram of wet cell mass and frozen at -80 C. Roughly 5-7 g of wet mass per liter of media is generally obtained.

After thawing, cells were lysed through sonication using a qSonica Q500 probe-tip sonicator with 1/8"-1/4" probes, dependent on sample volume, in 30 s on : 30 s off cycles and 25% amplitude until 3,000 kJ of input energy was reached. Cell suspension should turn a darker shade of brown as lysis increases. An additional 1 mM DTT was added after sonication. Sonicated lysates were transferred into 1.5 mL microcentrifuge tubes and centrifuged at 18,000 rcf for 10 min at 4 C and the supernatant transferred to fresh 1.5 mL microcentrifuge tubes. Lysates were incubated at 37 C for 30 min to perform the run-off reaction and centrifuged again at 10,000 rcf for 10 min at 4 C. Supernatant was transferred to fresh 1.5 mL microcentrifuge tubes before flash freezing and storage at -80 C.

CFPS reactions

Cell-free protein synthesis (CFPS) reactions (1 mL scale) were prepared in 1.5 mL microcentrifuge tubes by combining lysate (25% final reaction volume), plasmid, and water with the following components, based on the widely used PANOx-SP system[1] with slight modifications: 1.2 mM ATP; 0.86 mM GTP, UTP, and CTP; 34 µg/ml folinic acid; 170 µg/ml E. coli tRNA; 2 mM of each amino acid (-glutamic acid), 0.33 mM NAD; 0.27 mM Acetyl-CoA; 1.5 mM spermidine; 1 mM putrescine; 175 mM potassium glutamate; 10 mM ammonium glutamate; 2.7 mM potassium oxalate; 10 mM magnesium glutamate; and 33 mM PEP. A specified amount of pET-15b plasmid encoding His-Avi-tagged GFP DNA was used for each reaction. Anti-GFP antibody (Abcam 1218) was included at the specified concentrations. CFPS reactions were loaded onto coverslips for FCS tracking or, for microencapsulation, into glass syringes and kept on ice before connecting to syringe pumps and microfluidic device.

Sample slide preparation

Sample slides consisted of an imaging spacer with wells containing the sample solutions sandwiched by two glass coverslips. In initial tests, an imaging spacer 120 µm in thickness with circular well cutouts (Grace BioLabs #470352) was used. The spacer, which had adhesive on both sides, was first adhered to a glass coverslip. After pipetting 2-3 µl of the sample solution (cell-free reaction) into the center of each 6.35 mm-diameter well, the wells were sealed by

adhering a second coverslip over the spacer. The solution formed a ~3 mm area bounded on the top and bottom by the two coverslips and did not fill up the entire area of the well (Figure S1a). The sample slide was placed on the FCS objective for data acquisition. We observed the fluorescent signal in each well had a radial spatial gradient, with high signal near the edge of the solution and low signal near the center (Figure S1b). This spatial gradient was only observed for cell-free production of GFP; the signal was uniform for purified GFP protein and fluorescent dyes.

We hypothesized that the radial spatial gradient was due to availability of oxygen because the edge of the cell-free reaction solution was in contact with air left in the wells, and oxygen is required for both cell-free protein production and GFP maturation. Therefore, we switched to a thicker 1.8 mm spacer with 3 mm-diameter wells (Grace BioLabs #666208). To provide uniform accessibility to air, we filled the bottom half of the well with 6 µl cell-free reaction, leaving a layer of air above the solution (Figure S1c). Now we found uniform signal in xy and z. We used these thicker spacers for all experiments except when measuring microcapsules, for which we used the thin 120 µm spacers to immobilize the capsules between two coverslips.

Samples were kept on ice before slide preparation, and slides were kept on ice before starting data acquisition on the FCS instrument.

Microencapsulation device fabrication

The microcapillary device was fabricated based on prior work [2,3]. Briefly, the base of the device is formed by bridging two 2 by 3-inch glass slides by epoxy and two small glass strips. A round glass capillary (15.24 cm long with an outer diameter of 1.0 mm and inner diameter of 0.580 mm, World Precision Instruments, Sarasota, FL) and a square capillary (with an internal width of 1.0 mm, VitroCom, Mountain Lakes, NJ) compose the main components of the device. The square capillary is glued to the base after being cut to the desired length. The round capillary is centered in a pipette puller (Model P-97, Sutter Instruments, Novato, CA) to decrease its diameter in the center under tension and heat, breaking into two equally tapered capillaries. The tapered glass capillaries were then cleaved to the desired final diameters using a microforge station (Micro Forge MF 830, Narishige, Japan). Typical inner diameters of the capillaries ranged from 20 µm to 300 µm. After cleaning in an ethanol solution with 10 minutes of sonication, tips were treated separately with different silane solutions to change the glass hydrophilicity and hydrophobicity. The inner fluid capillary tip is treated to be hydrophobic so that the aqueous inner fluid could be easily repelled to break up into drops at the end of the capillary. Similarly, hydrophilic coating was applied to the exit capillary to accelerate the breakup of the oil-based middle fluid.

Microencapsulation

Syringe pumps (Harvard PHD Ultra, Harvard Apparatus, Holliston, MA) are used to pump inner (core), middle (shell), and outer (continuous phase) into the devices. Because of the use of biological sample, the syringe containing core fluid is usually cooled with an ice bag wrapped

around the syringe. Once the device is filled with liquid, flow rates are adjusted for obtaining stable double emulsion drop formation. After drops exited the device, they continued to travel to a UV-crosslinking section where a 365 nm UV lamp (UVP Multiple-Ray Lamp, Fisher Scientific, Hampton, NH) crosslinked the polymer in the shell phase, producing microcapsules. Glass vials were typically used for capsule collection by inserting the end of the exit tubing into the vial prefilled with 10ml of PBS solution. Emulsion drops are typically collected for a period of 15-20 mins, depending on the need. Then the continuous fluid was replaced by using filtration tool to remove the original continuous fluid and resuspend the capsules in fresh solution containing 10 wt% glycerol and 2 wt% poly vinyl alcohol for osmotic balancing with the core fluid.
Drop production was visualized on a microscope equipped with a fast camera (Photron Mini AX100, Photron, San Diego, CA) capable of up to 540,000 frames/sec. Images of the double emulsion drops and capsules were analyzed with the freely available image analysis program, ImageJ (Rasband, W.S., U. S. National Institutes of Health, Bethesda, MD).

FCS instrument

The FCS instrument was homebuilt using a multiple wavelength (637 nm, 561 nm, 488 nm, 405 nm) laser system (Stradus VersaLase 4, Vortran Laser); the laser is coupled through an optical fiber and used for excitation. A photodiode (PDA-155, Thorlabs) is used to monitor the laser power. An excitation DM (Di01-R405/488/561/635-25x36, Semrock) reflects excitation wavelengths and transmits emission wavelengths. We use a 60x water immersion objective (UPlanApo/IR, 1.20 NA, Olympus) to focus the excitation beam into a detection volume of ~1.6 fL. The emission beam is directed through a 50 mm pinhole and split into red and green wavelengths by the emission DM (FF562-Di03-25x36, Semrock). The split beam then passes through bandpass filters (FF01-609/54-25 and FF02-525/40-25, Semrock) and is split again by 50/50 beamsplitters onto two avalanche photodiodes. We use four APD detectors (PD-050-CTD, Micro-Photon-Devices), two per color, for detection of photon counts. For this work, we used the 488 nm laser and the two APDs in the green emission channel.

The detection volume was determined by a calibration using solutions of Atto 488 dye at known concentrations. The concentration of a stock solution of Atto 488 was measured using a spectrometer (Nanodrop), and dilutions ranging from 500 pM to 100 nM were made from the stock then measured on FCS. Correlations were fitted to the 1-species model. The volume was calculated as the slope of a linear fit to the plot of inverse amplitude vs. concentration. The measured detection volume ranged from 1.5-1.8 fL. This variation could be due to slight differences in alignment and environmental factors (e.g. temperature) over time.

FCS data acquisition

Using homebuilt software, arrival times of each photon were recorded on a counter card (National Instruments PCIe-6612). There are three relevant acquisition time parameters: 1. The duration of one “segment” of photon arrival times (30 s in this paper), from which one correlation curve is determined; 2. The frequency of acquiring segments, or the time between

acquisitions for one sample (~5-15 min in this paper); and 3. The total experimental time for which data was acquired (~2-10 hr in this paper). We explain each time parameter below.

First, 30 seconds was sufficient for good correlations as judged by the consistency of the resulting fitted parameters with repeat experiments. Specifically, 30 s of data was sufficient for obtaining good binding curves in the control titration experiments. We had also used 1 min but the slight increase in signal-to-noise in the correlation curves and fitting parameters did not appreciably change results. Acquisition times shorter than 30 s may also work, however this was not explored or characterized since 30 s sufficed for our purposes.

Second, the frequency of 30-s measurements needs to accurately capture the changes in concentration and binding due to protein expression. In theory, the upper limit of the frequency is determined by the acquisition time (30 s) and the number of sample wells to cycle through. For example, if we have an experiment that tracks 6 sample wells (eg. 1. Rb lysate alone, 2. GFP plasmid in Rb, 3. GFP plasmid and antibody in Rb, 4. CC lysate alone, 5. GFP plasmid in CC, 6. GFP plasmid and antibody in CC), then one full cycle consisting of 6 segments of 30-s acquisitions would be 3 minutes, neglecting moving time between samples. This means the interval between data acquisitions on the same sample is 3 min. In practice, we allow for moving and settling time between samples and sometimes also decrease the frequency to reduce the data file size while still acquiring data often enough to track protein synthesis. The data in this paper have intervals of ~5-15 min between the same sample.

Third, for time series data tracking GFP synthesis, the length of time that 30-s segments were repeatedly acquired ranged from ~2-10 hr, depending on the needs of the experiment. For example, some experiments tracked expression to ~10 hr to demonstrate the full temporal expression profile from start to past saturation (eg. Figures 2 and S2), while others tracked only ~2 hr as that sufficed for a binding curve (eg. Figure 4C).

FCS data analysis

Correlations were calculated following the method outline in Reference [4]. The cross correlation between the two green APDs was used as the autocorrelation function to avoid the afterpulsing effect.

Correlations were fitted to 1- or 2-species models to obtain amplitudes and diffusion times. Error bars on time series plots of amplitudes, concentrations, and diffusion times represent fitting errors. Each data point on the time series plots of photon count was calculated by total photons divided by measurement time (30 s).

For fits to correlation data with two species, we first fitted to the 1-species model to confirm the overall trend in diffusion time. Then the correlation data was fitted to the 2-species model with the two diffusion times τ_{D1} and τ_{D2} fixed at values indicated by the 1-species fit (the unbound and fully bound diffusion times). Typically, unbound τ_{D1} = 0.23 ms and bound τ_{D2} = 0.46 ms in homemade lysate and buffer; τ_{D1} = 0.35 ms and bound τ_{D2} = 0.76 ms in commercial lysate. Slight

variations in measured diffusion times could be due to variations in detection volume/alignment. We noted earlier that the measured diffusion time of GFP is higher in the commercial lysate than in homemade lysate or buffer, perhaps due to interactions with components of the lysate or oligomerization in the buffer condition of the lysate. Thus, batch variations in the commercial lysate could also contribute to variations in measured τ_{D1} and τ_{D2}.

Derivation of the quadratic binding equation

Equations for steady state equilibrium binding can be derived from first principles and are presented in various biochemistry textbooks, for example in Segel 1975 (ref [5]).

For two species binding in solution,

$$A + B \leftrightarrow AB$$

their dissociation constant is defined at equilibrium as

$$K_D = \frac{[A][B]}{[AB]} \qquad \text{Equation S1}$$

where [*A*], [*B*], and [*AB*] are the concentrations of the free and bound species at equilibrium. In general in any experiment, we know or dictate the total concentrations $[A]_{\text{tot}}$ and $[B]_{\text{tot}}$, which can be expressed as

$$[A]_{\text{tot}} = [A] + [AB] \qquad \text{Equation S2}$$

$$[B]_{\text{tot}} = [B] + [AB] \quad . \qquad \text{Equation S3}$$

To express [*AB*] as a function of $[A]_{\text{tot}}$, $[B]_{\text{tot}}$, and K_D, rearrange and substitute Equations S2 and S3 into Equation S1:

$$K_D = \frac{([A]_{\text{tot}} - [AB])([B]_{\text{tot}} - [AB])}{[AB]} \quad . \qquad \text{Equation S4}$$

Rearrange Equation S4 to obtain

$$0 = [AB]^2 - ([A]_{\text{tot}} + [B]_{\text{tot}} + K_D)[AB] + [A]_{\text{tot}}[B]_{\text{tot}} \quad . \qquad \text{Equation S5}$$

This is a quadratic equation in [*AB*], and the solution is given by

$$[AB] = \frac{[A]_{\text{tot}} + [B]_{\text{tot}} + K_D - \sqrt{([A]_{\text{tot}} + [B]_{\text{tot}} + K_D)^2 - 4[A]_{\text{tot}}[B]_{\text{tot}}}}{2} \qquad \text{Equation S6}$$

where we only kept the negative root since [*AB*] must equal 0 if $[A]_{\text{tot}} = 0$ or $[B]_{\text{tot}} = 0$. Finally, the fraction of *B* bound is given by

$$f_{\text{B}_{\text{bound}}} = \frac{[AB]}{[B]_{\text{tot}}}$$

$$= \frac{[A]_{\text{tot}} + [B]_{\text{tot}} + K_D - \sqrt{([A]_{\text{tot}} + [B]_{\text{tot}} + K_D)^2 - 4[A]_{\text{tot}}[B]_{\text{tot}}}}{2[B]_{\text{tot}}} \quad . \qquad \text{Equation S7}$$

Note that when $[B]_{tot}$ << $[A]_{tot}$ and K_D (B is in trace amount), Equation S7 reduces to the simple hyperbolic binding equation ($f_{B_{bound}} = \frac{[A]_{tot}}{[A]_{tot}+K_D}$), as derived by a Taylor expansion of Equation S7 or by repeating the above derivation using $[A]_{tot} = [A]$.

Equation S7 is the quadratic binding Equation 6 in the main text with $[B]_{tot} = [G]$ (total GFP concentration) and $[A]_{tot} = [a]$ (total antibody concentration). When $[G]$ was held constant and $[a]$ varied in control experiments, $f_{GFP_{bound}}$ was plotted as a function of $[a]$, and Equation 6 was fitted to the data with the parameters K_D and $[G]$. When $[a]$ was held constant and $[G]$ varied in control and time series experiments, $f_{GFP_{bound}}$ was plotted as a function of $[G]$, and Equation 6 was fitted to the data with the parameters K_D and $[a]$.

Additional Results and Discussion

Tuning the rate and amount of protein synthesis by plasmid concentration

Accurate measurements of protein binding affinity require appropriate regimes of the concentrations of binding partners [6]. Ideally, the concentration of one binding partner (the "trace" species) should be much lower than the expected K_D while the concentration of the other partner should be varied to capture the full range of fraction of the trace species bound. In any assay, a practical constraint to how low in concentration the trace species can be, as needed to measure very low K_Ds, is the lowest detectable signal above background. While picomolar concentrations of GFP (and thus K_Ds) are measurable in our FCS system when using buffer, the detection limit rises to low nanomolar in the background of cell-free lysate, thus also raising the lowest measurable K_Ds to the low nanomolar range. Furthermore, in our approach to using time resolved protein synthesis as the concentration variable, the rate and amount of synthesis must be tuned to achieve the full range of fraction bound. If the protein is made too quickly, the low-concentration region of the binding curve may not be captured, whereas if the protein is made too slowly, the cell-free reaction may stop before reaching a final yield high enough to capture the high concentration region.

Thus, to optimize the synthesis rate and yield for binding measurements and to further characterize the commercial and homemade lysates, we measured the production of GFP using different amounts of plasmid (Figure S2). While much higher plasmid concentrations (~10-15 µg/ml, or 2.3-3.5 nM) are typically used in cell-free reactions to achieve high yield, we tested a lower range (0.5-5 µg/ml, or 0.12-1.2 nM) because slower protein synthesis better captures the low concentrations required for measuring the expected low K_D between GFP and its antibody. As expected, higher plasmid concentrations resulted in faster rates and higher final yields. 5 µg/ml plasmid produced ~140 nM GFP within 5 hr at room temperature (Figure S2a). Using 10x less plasmid (0.05 µg/ml in the homemade lysate), our FCS system was still able to measure a clear signal above lysate background with clean correlations, plateauing at ~15 nM GFP in 5 hr (Figure S2b). Diffusion times of expressed GFP (τ_D ~0.3 ms and ~0.2 ms in commercial and homemade lysates, respectively) were independent of plasmid concentration and remained

constant over time, as expected. Overall, the homemade lysate had slightly slower production rates than the commercial lysate but similar final yields.

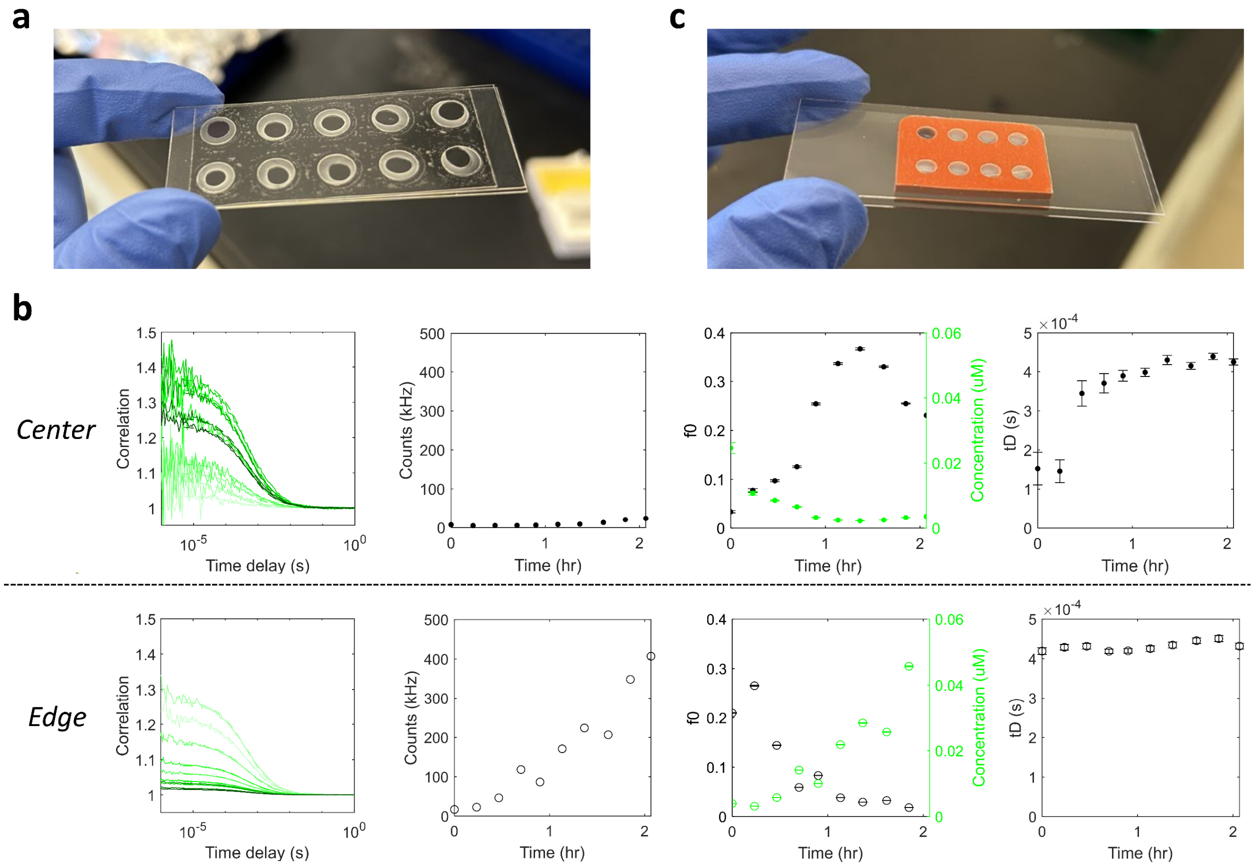

Figure S1. Sample chambers must provide sufficient oxygen for cell-free production of fluorescent proteins. a. A sample slide consisting of wells ~120 um deep and 6.35 mm wide sealed between two coverslips. 2 µL of sample solution was deposited into each well, which formed a patch ~3 mm in width (they appear as the dark circles inside each clear well) surrounded by residual air inside each well. **b.** FCS time series data acquired near the center (top) and edge (bottom) of the same well of a slide prepared as in **(a)**, demonstrating significantly faster and higher increase of GFP signal near the edge than near the center. 1 µg/ml GFP plasmid in commercial cell-free lysate was used for this data. Near the center, the GFP signal remained low for over 1 hr before rising slowly; correlation curves started out noisy with low amplitude since the signal was dominated by the lysate background; as GFP was slowly produced, the amplitude increased initially as the signal rose above lysate background, then decreased as the GFP concentration further increased; and the fitted diffusion times slowly increased from a value corresponding to the noisy lysate background to the value corresponding to GFP. In contrast, the GFP signal near the edge of the solution patch increased much faster, presumably because oxygen was more readily available near the border with air. **c.** A sample slide consisting of wells ~1.8 mm deep and 3 mm wide between two coverslips. To provide uniform accessibility to air, we filled the bottom half of these wells with 6 µl cell-free reaction, leaving a layer of air above the solution. This configuration resulted in uniform GFP signal in all directions.

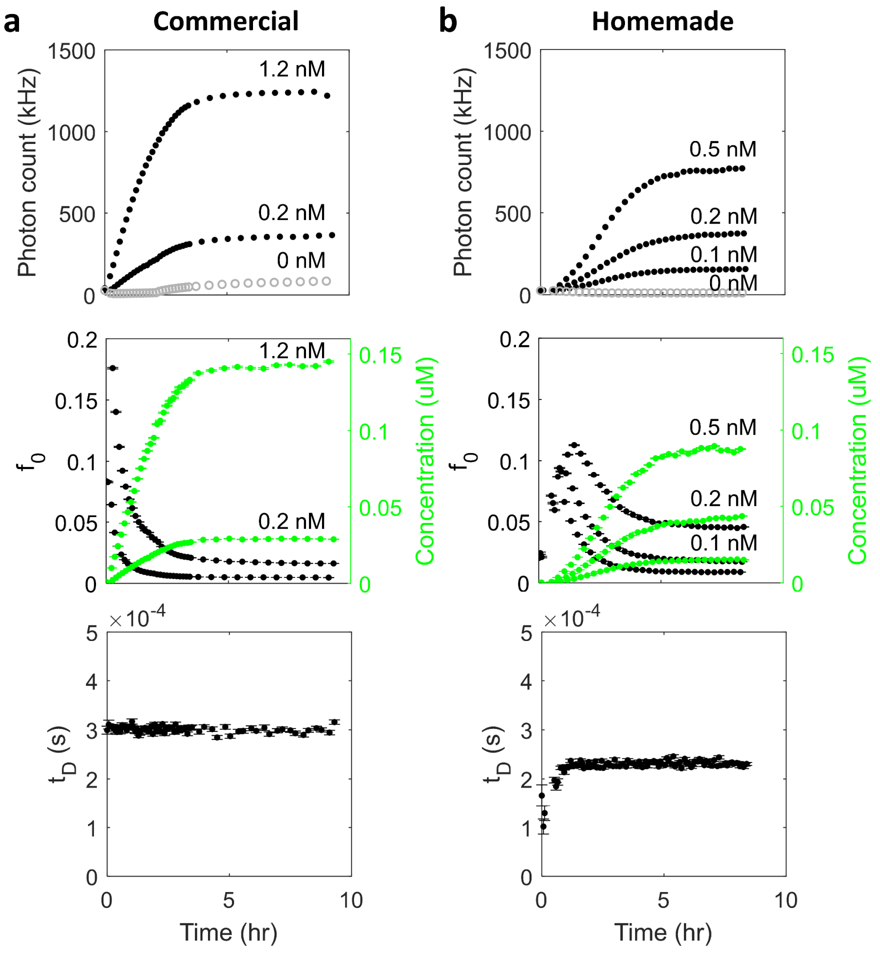

Figure S2. Rate and amount of protein synthesis can be tuned by plasmid concentration. a-b. Photon count rate (top), fitted amplitude f_0 (middle) and diffusion times t_D (bottom) are plotted over experimental time for several GFP plasmid concentrations in commercial (1.2 and 0.2 nM) **(a)** and homemade (0.1, 0.2, and 0.5 nM) **(b)** lysates. Lysate-only photon count rates are plotted as gray open circles. Calculated concentrations included background subtraction. The homemade lysate had slightly slower production rates than the commercial lysate but similar final yields.

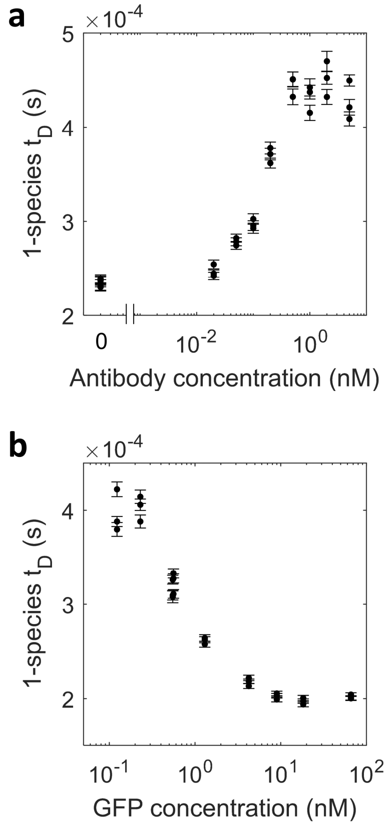

Figure S3. One-species diffusion times of GFP in binding measurements by titration. a. When GFP concentration was held constant (200 pM in this case) and antibody concentration titrated, diffusion times from one-species fits (Equation 1) to correlations increased as a function of antibody concentration. **b.** When antibody concentration was held constant (400 pM in this case) and GFP concentration titrated, diffusion times decreased as a function of GFP concentration. These are one-species diffusion times to illustrate the shift in overall mass upon binding. To calculate the fraction bound (as plotted in Figures 3-6), we fitted the two-species model to correlations (Equations 4 and 5).

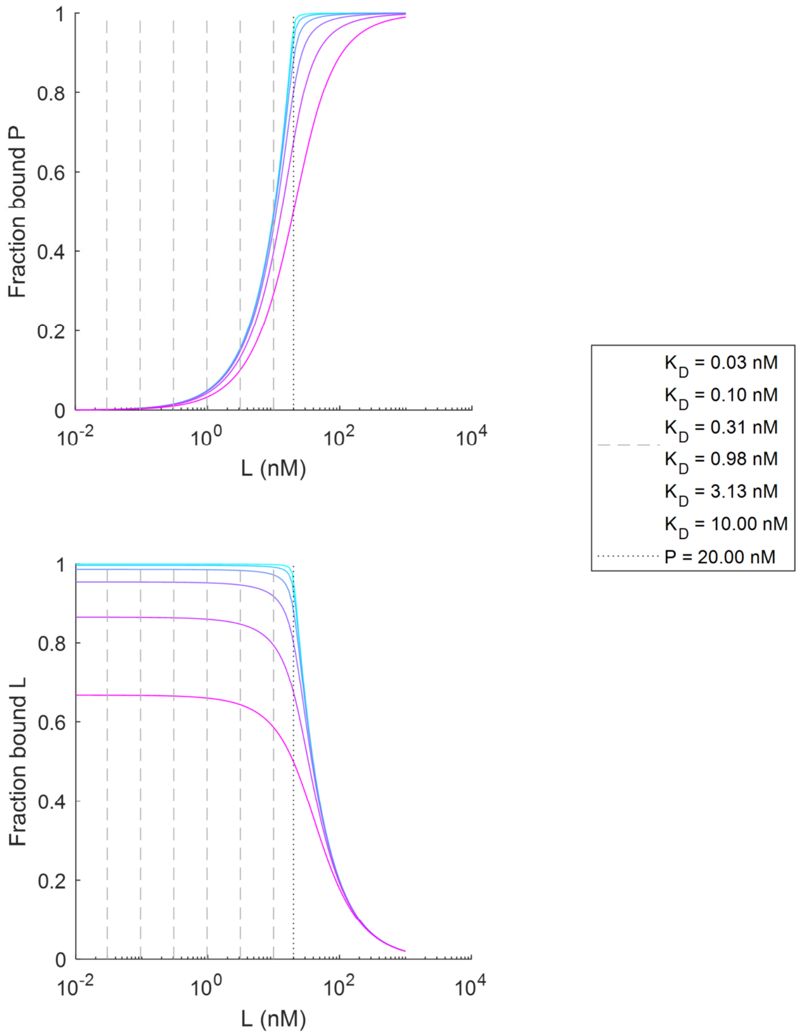

Figure S4. Accurate determination of K_D is not possible when the trace protein concentration is much higher than the true K_D, as illustrated by simulated binding curves. In this example, the trace protein concentration is set to P = 20 nM, and binding curves corresponding to K_Ds ranging from 30 pM (cyan) to 10 nM (magenta) are simulated. Dashed lines represent the K_D values, and the dotted line represents the P value, for comparison. The top panel plots fraction of the trace protein bound as a function of ligand concentration (the canonical way of plotting a binding curve), and the bottom plots fraction of ligand bound as a function of ligand concentration. It is apparent that the binding curves corresponding to the lower picomolar K_Ds would be indistinguishable from each other in the presence of experimental noise, as expected since P = 20 nM is much higher than the K_Ds. In these cases, the measured K_D would be interpreted as the upper bound.

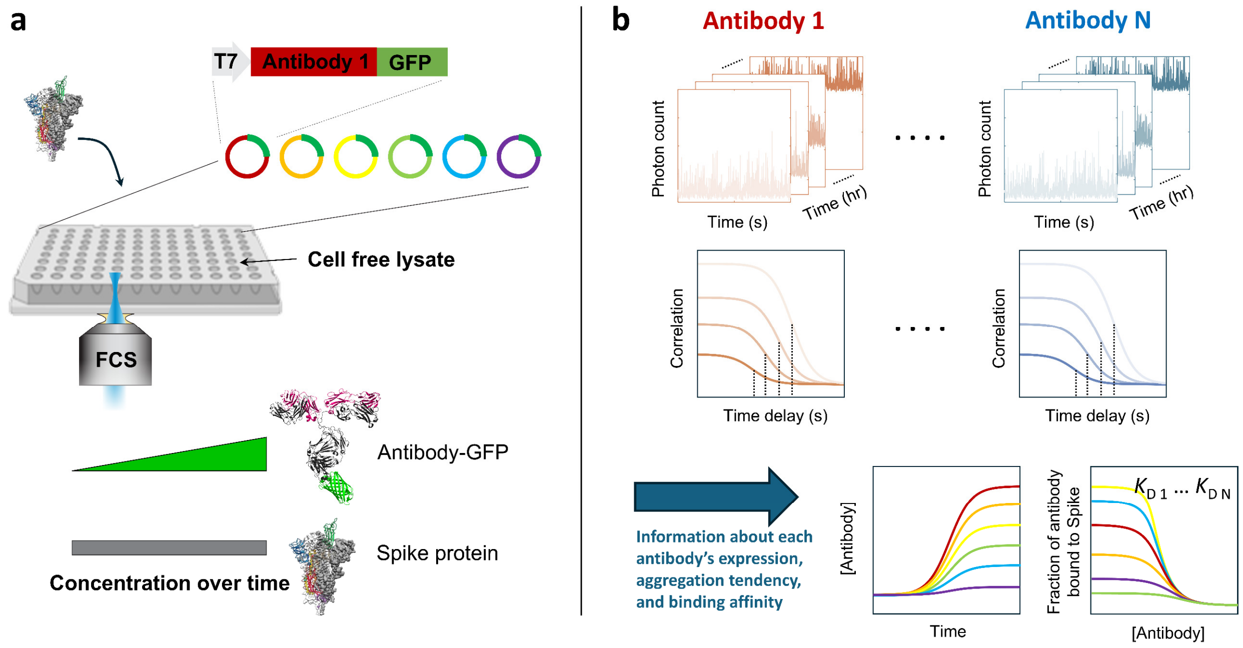

Figure S5. Approach for high throughput screening of candidate binders using the "one-pot reaction" method. a. In this illustration, different antibody or antibody fragment constructs are to be screened for binding to the COVID spike protein. GFP is a part of the antibody construct and provides the fluorescence signal for FCS. Each sample well contains the cell-free lysate, the purified spike protein, and a plasmid encoding one antibody-GFP candidate. FCS data is acquired in each well over cell-free expression time so that the concentrations of antibody-GFP increase while the concentration of the spike protein remains constant, analogous to the experiments presented in this paper in which GFP was expressed over time as the anti-GFP antibody remained constant. **b.** Overview of data analysis. Each candidate antibody would have a set of FCS data, e.g., 30 s of photon stream repeatedly acquired over 5 hr. Each 30 s segment of photon stream would yield a correlation curve from which the concentration of the antibody-GFP and the fraction bound are obtained. From here, each antibody would have an expression profile (concentration over time) and a binding curve.

Supporting Information References

1 Jewett, M. C. & Swartz, J. R. Mimicking the Escherichia coli cytoplasmic environment activates long-lived and efficient cell-free protein synthesis. *Biotechnol Bioeng* **86**, 19-26 (2004). https://doi.org:10.1002/bit.20026

2 Ye, C., Chen, A., Colombo, P. & Martinez, C. Ceramic microparticles and capsules via microfluidic processing of a preceramic polymer. *J R Soc Interface* **7 Suppl 4**, S461-473 (2010). https://doi.org:10.1098/rsif.2010.0133.focus

3 Utada, A. S. *et al.* Monodisperse double emulsions generated from a microcapillary device. *Science* **308**, 537-541 (2005). https://doi.org:10.1126/science.1109164

4 Laurence, T. A., Fore, S. & Huser, T. Fast, flexible algorithm for calculating photon correlations. *Opt. Lett.* **31**, 829-831 (2006). https://doi.org:10.1364/OL.31.000829

5 Segel, I. H. *Enzyme kinetics; behavior and analysis of rapid equilibrium and steady-state enzyme systems*. (Wiley, 1975).

6 Jarmoskaite, I., AlSadhan, I., Vaidyanathan, P. P. & Herschlag, D. How to measure and evaluate binding affinities. *eLife* **9**, e57264 (2020). https://doi.org:10.7554/eLife.57264
